# *Verticillium dahliae* chromatin remodeling facilitates the DNA damage repair in response to plant ROS stress

**DOI:** 10.1371/journal.ppat.1008481

**Published:** 2020-04-16

**Authors:** Sheng Wang, Xue-Ming Wu, Chuan-Hui Liu, Jing-Yun Shang, Feng Gao, Hui-Shan Guo

**Affiliations:** 1 State Key Laboratory of Plant Genomics, Institute of Microbiology, Chinese Academy of Sciences, Beijing, China; 2 CAS Center for Excellence in Biotic Interactions, University of the Chinese Academy of Sciences, Beijing, China; 3 Shenzhen Baoan Women’s and Children’s Hospital, Jinan University, Shenzhen, China; University of Dundee, UNITED KINGDOM

## Abstract

Reactive oxygen species (ROS) production is one of the earliest responses when plants percept pathogens and acts as antimicrobials to block pathogen entry. However, whether and how pathogens tolerate ROS stress remains elusive. Here, we report the chromatin remodeling in *Verticillium dahliae*, a soil-borne pathogenic fungus that causes vascular wilts of a wide range of plants, facilitates the DNA damage repair in response to plant ROS stress. We identified *VdDpb4*, encoding a histone-fold protein of the ISW2 chromatin remodeling complex in *V*. *dahliae*, is a virulence gene. The reduced virulence in wild type Arabidopsis plants arising from *VdDpb4* deletion was impaired in the *rbohd* mutant plants that did not produce ROS. Further characterization of VdDpb4 and its interacting protein, VdIsw2, an ATP-dependent chromatin-remodeling factor, we show that while the depletion of *VdIsw2* led to the decondensing of chromatin, the depletion of *VdDpb4* resulted in a more compact chromatin structure and affected the VdIsw2-dependent transcriptional effect on gene expression, including genes involved in DNA damage repair. A knockout mutant of either *VdDpb4* or *VdIsw2* reduced the efficiency of DNA repair in the presence of DNA-damaging agents and virulence during plant infection. Together, our data demonstrate that VdDpb4 and VdIsw2 play roles in maintaining chromatin structure for positioning nucleosomes and transcription regulation, including genes involved in DNA repair in response to ROS stress during development and plant infection.

## Author summary

ROS production is one of the earliest responses after the perception of pathogen-associated molecular patterns by plant transmembrane immune receptors, and dependent on the respiratory burst oxidase homolog (RBOH). ROS cause DNA oxidative damage and acts as antimicrobials to block pathogen entry. In this study, we found that chromatin remodeling components, including VdDpb4 and its interacting protein, VdIsw2, are essential for the *V*. *dahliae* tolerant in response to ROS stress during development and plant infection. Assays of the accessibility of bulk chromatin suggest that VdDpb4 plays an important role in maintaining a more “open” and accessible chromatin landscape, while VdIsw2 plays an antagonistic role in balancing chromatin structure. Abnormality of nucleosome repositioning by depletion of either protein is harmful to the fungus during DNA repair in response to ROS stress during development and plant infection. We further found that VdDpb4 is required for VdIsw2 to bind to gene promoters for appropriate RNA polymerase II transcription. Taken together, our data demonstrate that VdDpb4 is required for the location of ISW2 on DNA and VdIsw2-dependent transcriptional regulation of gene expression; and provide the first example and essential information for further investigation of chromatin-associated complexes in pathogenic fungi.

## Introduction

Pathogen recognition induces the production of reactive oxygen species (ROS) by NADPH oxidases in both plants and animals [1]. ROS production is one of the earliest responses after the perception of pathogen-associated molecular patterns (PAMPs) by plant transmembrane immune receptors, and dependent on the respiratory burst oxidase homolog (RBOH) [2, 3]. In addition to act as local and systemic secondary messengers to trigger immune responses, ROS have been proposed to act as antimicrobials, cross-linkers of the plant cell wall to block pathogen entry [1]. However, whether and how pathogens tolerate ROS stress remains elusive.

ROS cause DNA oxidative damage, such as modified nucleotide bases or single-strand breaks, that are corrected by the base-excision repair (BER) pathway [4]. Other DNA repair pathways, such as double-strand break (DSB) repair pathways, nucleotide-excision repair (NER), and mismatch repair (MMR), have also been reported to be involved in ROS-induced DNA damage in yeast and mammalian cells [5]. The genomic DNA packaged inside chromatin hinders DNA accessibility and its subsequent repair [5]. The basal units of chromatin are nucleosomes, which consist of ~145–147 bp of DNA wrapped around a histone octamer that contains 2 copies of each histone (H2A, H2B, H3 and H4). Alteration in the relative positions of nucleosomes and DNA by chromatin remodeling plays a significant role in regulating diverse biological processes in cells [6]. Chromatin remodeling complexes are ATP-dependent enzymes that facilitate the movement of histones relative to DNA, leading to a condensing or decondensing of chromatin, and control the chromatin structure and assembly to regulate gene expression spatially and temporally [7].

The interplay between chromatin remodelers and the DNA repair pathways have recently been elucidated. For example, in vitro repair of UV-damaged reconstituted nucleosomes through photoreactivation by DNA photolyases is facilitated by the ATP-dependent remodelers SWI/SNF by remodeling the damaged nucleosome [8]. In yeast, the major DSB repair pathways require histone H2A phosphorylation surrounding a DSB to allow the binding of factors that influence chromatin structure, such as ATP-dependent remodelers, histone modifying enzymes and chromatin assembly factors, as well as recruitment of DNA repair factors [5, 9, 10].

Four different families of chromatin remodeling complexes are characterized by their unique domain residing within or adjacent to the ATPase domain: SWI/SNF family, ISWI family, CHD family, and INO80 family [7]. All four complexes share a similar ATPase domain and have the ability to alter histone-DNA interactions by utilizing ATP hydrolysis. The ISWI (imitation switch) family remodelers were first reported in *Drosophila melanogaster* and later demonstrated to be conserved in humans and yeast. To date, 7 different ISWI complexes in humans and 2 different ISWI complexes in yeast have been described [6, 7].

In this work, through the pathogenicity-deficient screening a T-DNA insertion library of *Verticillium dahliae*[11], a soil-borne fungal pathogen that infects over 400 plant species, including economically important crops, such as cotton and tomato [12], we identified Dpb4 in *V*. *dahliae*, one homologous component of the yeast ISW2 complex, named VdDpb4, that contributes to virulence during plant infection. The reduced virulence in wild type Arabidopsis plants arising from *VdDpb4* deletion was impaired in the *rbohd* mutant plants that did not produce ROS. Further analysis showed that the *V*. *dahliae* ISW2 complex, possibly containing VdDpb4, VdIsw2 and VdItc1, played roles in maintaining chromatin structure and transcription regulation, including genes involved in DNA repair. Depletion of VdDpb4 or VdIsw2 altered nucleosome positioning, accumulated much more damaged DNA in cells and disrupted the repair efficiency; the mutant strains developed abnormal morphologies and reduced virulence in host plants. Together with the ATPase domain of VdIsw2 required for the fungal virulence in plants, our data established the molecular features of the ISW2 chromatin remodeling complex and the effects of nucleosomes on DNA repair processes essential for the *V*. *dahliae* tolerant toward host ROS stress.

## Results

### VdDpb4 is required for the development and host infection of *V*. *dahliae*

**Fig 1:**
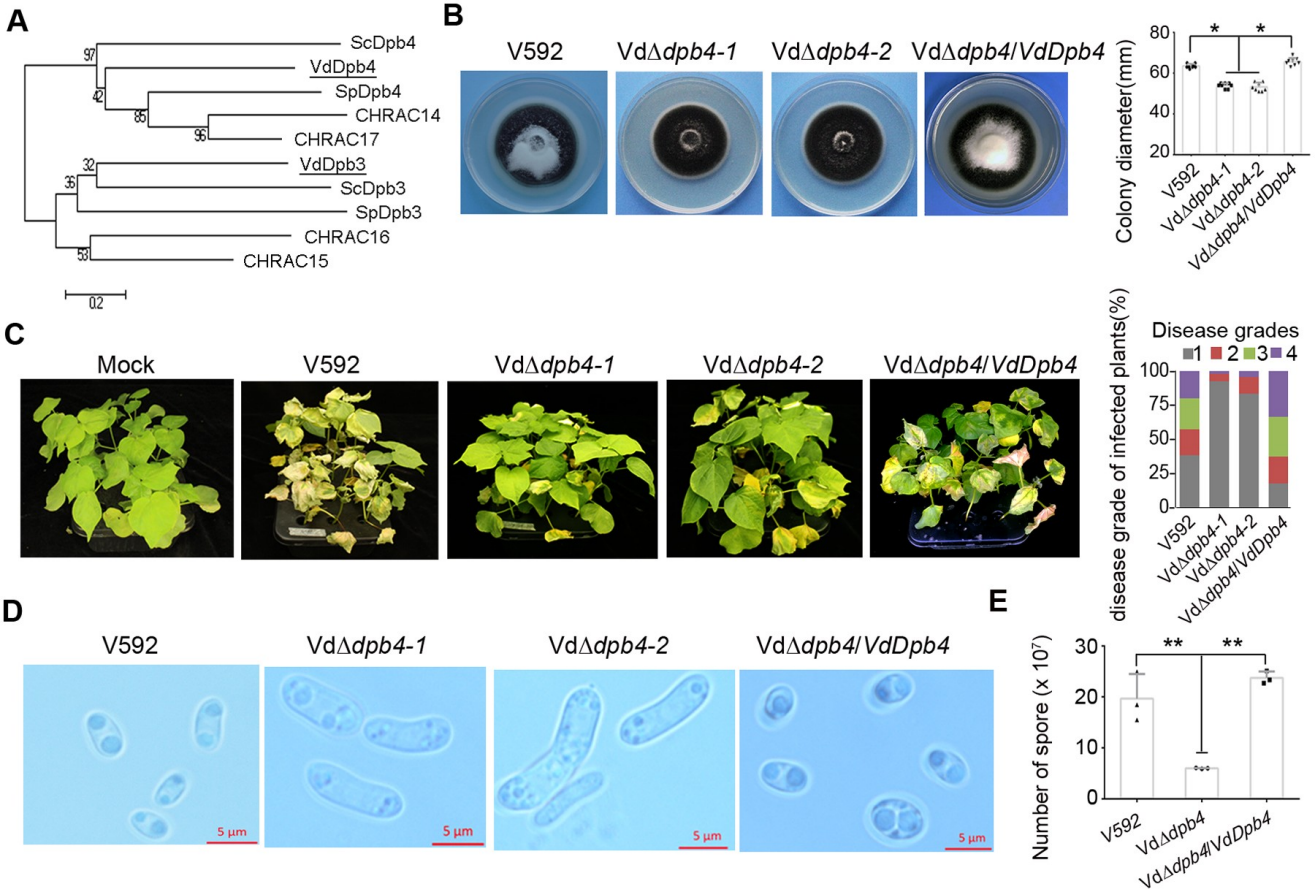
VdDpb4 is required for the development and host infection of *V*. *dahliae*. A. Phylogenic analysis of the VdDbp4 homologs with other Dpb4 proteins from yeast, *Drosophila* and humans. VdDbp4 and VdDpb3 are labeled. The analysis was performed using the neighbor-joining method phylogeny test with the bootstrap method (*No*. of bootstrap replications = 1000). GenBank accession number: *V*. *dahliae* Dpb4 (EGY18447.1) and VdDpb3 (EGY13855.1), *S*. *cerevisias* Dpb4 (NP_010406.3) and Dpb3 (NP_009837.1), *S*. *pombe* Dpb4 (NP_595521.1) and Dpb3 (NP_593726.1), *Drosophila* CHRAC14 (Q9V444.1) and CHRAC16 (NP_001285148.1), and humans CHRAC15 (NP_059140.1) and CHRAC17 (Q9NRF9.1). B. Colony morphology of wild-type V592, the VdΔ*dpb4* mutant strains, and the VdΔ*dpb4/VdDpb4* complementation strains on PDA plates 2 weeks postincubation. Quantification of colony diameter is shown on the right. Asterisks indicate significant differences (*P*<0.05; t-test, mean ± SD). C. Disease symptoms of cotton plants infected with the V592, mutant and complementation strains at 30 days postincubation (dpi). Disease grades on cotton leaves were classified into four levels depending on the ratio of (wilted and dropped off leaves / total leaves) during fungal invasion: grade 1 = 0–25%; grade 2 = 26–50%; grade 3 = 51–75%; and grade 4 = 76–100%. D. Microscopic observation of conidial morphology. E. Quantification of conidial production. Asterisks indicate significant differences (*P*<0.05; t-test, mean ± SD).

We previously constructed a T-DNA insertion library from the virulent defoliating cotton isolate *V*. *dahliae*, V592 [11] and found one pathogenicity-deficient mutant in which a single copy of the T-DNA integrated into the promoter of a hypothetical protein sequence, VDAG_08781, with incomplete sequence information. With the recently updated genome database, we found that the T-DNA knockout gene contains 1092 base pairs (bp) and encodes a 364-amino acid, protein with high similarity to *Saccharomyces cerevisiae* Dpb4, named VdDpb4 (Fig 1A). By genome mining, we found that Dpb4-related genes containing the histone-fold domain and coiled-coil domain are also widely distributed, including in pathogenic fungi (S1A Fig), yet the biological function has not been investigated in pathogenic fungi. For further investigation, a knockout mutant of *V*. *dahliae* for *VdDpb4*, VdΔ*dpb4*, was generated using the homologous recombination method [13] and confirmed by DNA gel blotting and RT-qPCR analyses (S1B–S1D Fig). The VdΔ*dpb4* mutants exhibited slightly lower hyphal growth rates with intense melanized microsclerotia on potato dextrose (PDA) plates (Fig 1B), but exhibited markedly reduced virulence in cotton plants in contrast to the wild-type (WT) V592 and VdΔ*dpb4/VdDpb4* complementation strains (Fig 1C). These results show that the *VdDpb4* gene is essential for fungal virulence in cotton plants.

During initial root colonization of *V*. *dahliae*, a few fungal hyphae differentiate into infection structures, hyphopodia and penetration pegs, to breach the root surface [14, 15]. Cellophane membrane laid on minimal medium is usually used to induce infection structures in a *V*. *dahliae* hyphal penetration assay. We observed hyphal penetration and growth on medium for both the WT V592 and VdΔ*dpb4* mutant strains (S2A and S2B Fig), suggesting that reduced virulence in VdΔ*dpb4*-infected cotton plants was due to its growth defect and/or incapability to counter host defenses, rather than the failure to develop infection structures on cotton roots. We also observed that the morphology of conidiospores in VdΔ*dpb4* mutants became much longer than that in V592 and VdΔ*dpb4/VdDpb4* (Fig 1D), and the sporulation of VdΔ*dpb4* mutants in liquid medium decreased to approximately 30% to that of WT V592 and complementation VdΔ*dpb4/VdDpb4* after 4 days of incubation (Fig 1E). Real-time quantitative PCR (RT-qPCR) analysis showed that the transcript level of *VdDpb4* was greatly induced at the early infection time point and constantly increased during WT V592 infection in cotton plants (S2C Fig). Taken together, our data demonstrate that *VdDpb4* is a virulence gene probably for the fungus that likely counters host defense during the initial colonization period and fungal development and sporulation during later infection processes, instead of incapable of melanized microsclerotial formation.

### VdDpb4 is essential for resistance to reactive oxygen species

**Fig 2:**
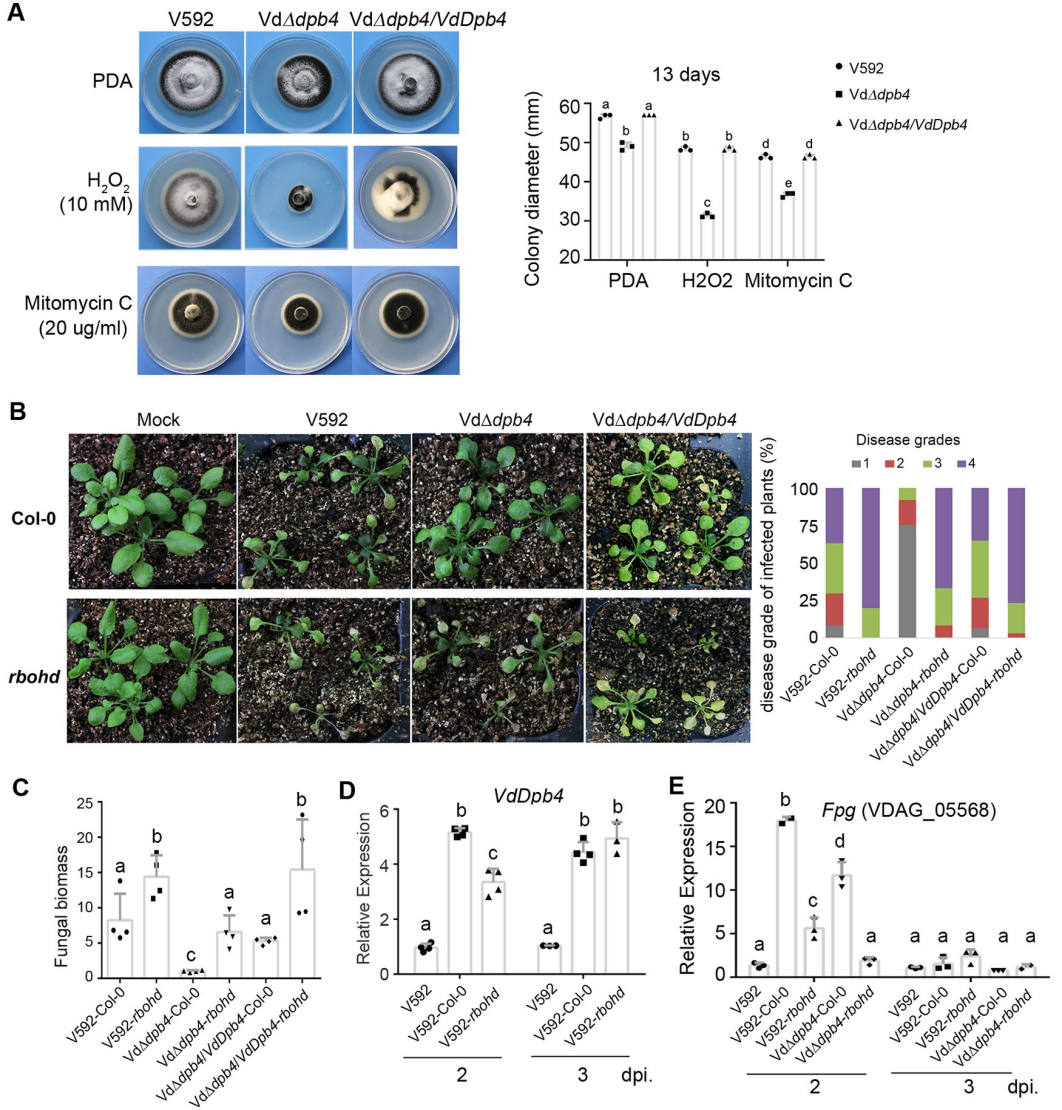
VdDpb4 is essential for resistance to reactive oxygen species. A. Mycelial growth on PDA agar medium in the presence of different DNA-damaging agents and quantification of colony diameter. Different letters indicate significant differences (*P*< 0.05, mean ± SD, one-way analysis of variance (ANOVA) followed by Tukey’s multiple comparisons test). B. VdDpb4 is essential for resistance to RBOHD-mediated defense. Disease symptoms of wild-type (Col-0) and *rbohd* mutant Arabidopsis plants infected with V592, mutant or complementation strains at 20 dpi. The disease grades were evaluated with three replicates of 48 plants for each inoculum. C. Reduced fungal biomass in VdΔ*dpb4*-infected Arabidopsis plants compared with V592-infected ones at 2-week postinoculation. The values were quantitative real time (qPCR) of fungal tubulin DNA relative to Arabidopsis At4g33380 DNA. Statistical analysis as described in A. D. Expression of *VdDpb4* was rapidly induced at 2- and 3-dpi during Arabidopsis plant infection as detected by reverse transcription qPCR (RT-qPCR). The value of *VdDpb4* mRNA relative to *elf1-α* at 2-dpi was arbitrarity designated as 1. Statistical analysis as described in A. E. Expression of *Fpg* was rapidly induced at 2-dpi during wild type Arabidopsis plant infection as detected by reverse transcription qPCR (RT-qPCR). Statistical analysis as described in A.

Pathogens need to overcome the stress conditions, including ROS and osmotic stress, during host penetration [16–18]. To test whether VdDpb4 is responsible for stress tolerance, the VdΔ*dpb4*, WT V592 and complementation VdΔ*dpb4/VdDpb4* strains were grown on medium plates containing various agents including hydrogen peroxide (H_2_O_2_), which was used to detect the tolerance to ROS, and sorbitol or NaCl, which were used to detect the tolerance to osmotic pressure. As shown in Fig 2A, VdΔ*dpb4* had a reduced growth rate in the medium containing 10 mM H_2_O_2_ to approximately 50% compared to the WT and complementation strains, whereas, retaining the intense melanized microsclerotial formation, VdΔ*dpb4* showed no significant differences in growth rate (colony diameter) compared to the WT on the media containing 0.6 M sorbitol or 0.5 M NaCl (S3A Fig). These results imply that VdDpb4 is mainly involved in the regulation of the oxidative stress response.

Many enzymes have been shown to play important roles in antagonizing hydrogen peroxide and its derivatives [19]. For instance, catalase catalyzes the breakdown of H_2_O_2_, and superoxide dismutase (SOD) exclusively scavenges harmful free radicals. RT-qPCR analysis showed that the transcript levels of catalase and SOD homologous genes were not reduced in the VdΔ*dpb4* mutant compared with WT V592, and some of the genes even increased in the VdΔ*dpb4* mutant strains (S3B and S3C Fig). Consistently, the enzyme activity of SOD was higher in VdΔ*dpb4* mutant than that in WT V592 (S3C Fig). These data suggest that the lower tolerance to H_2_O_2_ of the VdΔ*dpb4* mutant did not result from the down regulation of the catalase and SOD activity but the incapability to repair H_2_O_2_-induced DNA oxidative damage when *VdDpb4* was deleted. Indeed, upon treatment with other DNA-damaging agent, such as alkylating agent mitomycin C, the VdΔ*dpb4* mutants exhibited a growth reduction compared to WT V592, albeit in a lower extent compared to H_2_O_2_ treatment (Fig 2A), supporting that VdDpb4 plays a role mainly in DNA damage repair. Consistently, the expression levels of some genes involved in DNA damage repair pathways, such as the endonuclease/exonuclease/phosphatase family, formamidopyrimidine-DNA glycosylase (Fpg) and DNA repair protein rad50, significantly decreased in the VdΔ*dpb4* mutant compared to those in V592 (S3D Fig).

In plants, ROS production is predominantly dependent on the RBOH family member, RBOHD, which is the most highly expressed and plays important roles in cell death control, cell wall damage-induced lignification and systemic signaling in response to biotic and abiotic stresses [2]. Thus, the role of VdDpb4 in resistance to plant ROS effect was assessed in wild-type (Col-0) and *rbohd* mutant Arabidopsis plants with fungal infection. Compared with WT V592 and complementation VdΔ*dpb4/VdDpb4* strains, the VdΔ*dpb4* mutant displayed reduced virulence on the Col-0 plants (Fig 2B). The reduced virulence of the VdΔ*dpb4* was impaired in the *rbohd* mutant plants compared with that in the Col-0 plants (Fig 2B). Consistently, fungal biomass was significantly reduced in VdΔ*dpb4*-infected Col-0 but not in *rbohd* mutant plants compared with V592-infected ones (Fig 2C). Increased fungal biomass in V592-infected *rbohd* plants was also detected, supporting the RBOHD-mediated antifungal effect (Fig 2C). A great induction of *VdDpb4* transcript in V592-infected Col-0 plants was detected at 2-dpi (Fig 2D), while less induction of *VdDpb4* at 2-dpi but continuing to increase at 3-dpi was detected in V592-infected *rbohd* plants (Fig 2D), hinting that plant RBOHD-independent defense also induced *VdDpb4* expression with different mode in the absence of RBOHD-produced ROS. Taken together, these results suggest that *VdDpb4* is essential for resistance to RBOHD-produced ROS stress at early infection time point and possibly downstream defensive signaling.

To assess whether *V*. *dahliae* was exposed to RBOHD-produced ROS stress during early plant infection, we examined expression of *Fpg* gene at different infection time points. RT-qPCR analysis showed that the transcript level was significantly induced in V592-infected WT Col-0 plants compared to that in V592-infected *rbohd* mutant plants at 2 dpi; less induction was also detected in VdΔ*dpb4*-infected Col-0 but not in *rbohd* plants (Fig 2E), indicating that VdDpb4 plays role in countering RBOHD-produced ROS-caused fungal DNA damage at very early time point. Induced *Fpg* expression was not detected in plants of either genotype infected with WT or mutant fungal strains at 3 dpi (Fig 2E), in agreement with the fleetly ROS scavenging after *V*. *dahliae-*induced ROS burst in infected plants [20]. Taken together, we reason that VdDpb4 plays role in regulating genes involved in DNA damage repair in *V*. *dahliae* against RBOHD-dependent ROS stress during early infection. The results also suggest that, in addition to RBOHD-produced ROS, other stresses during infection would also induce DNA repair genes in a manner that might be or not dependent on VdDpb4.

### VdDpb4 is essential for DNA damage repair

**Fig 3:**
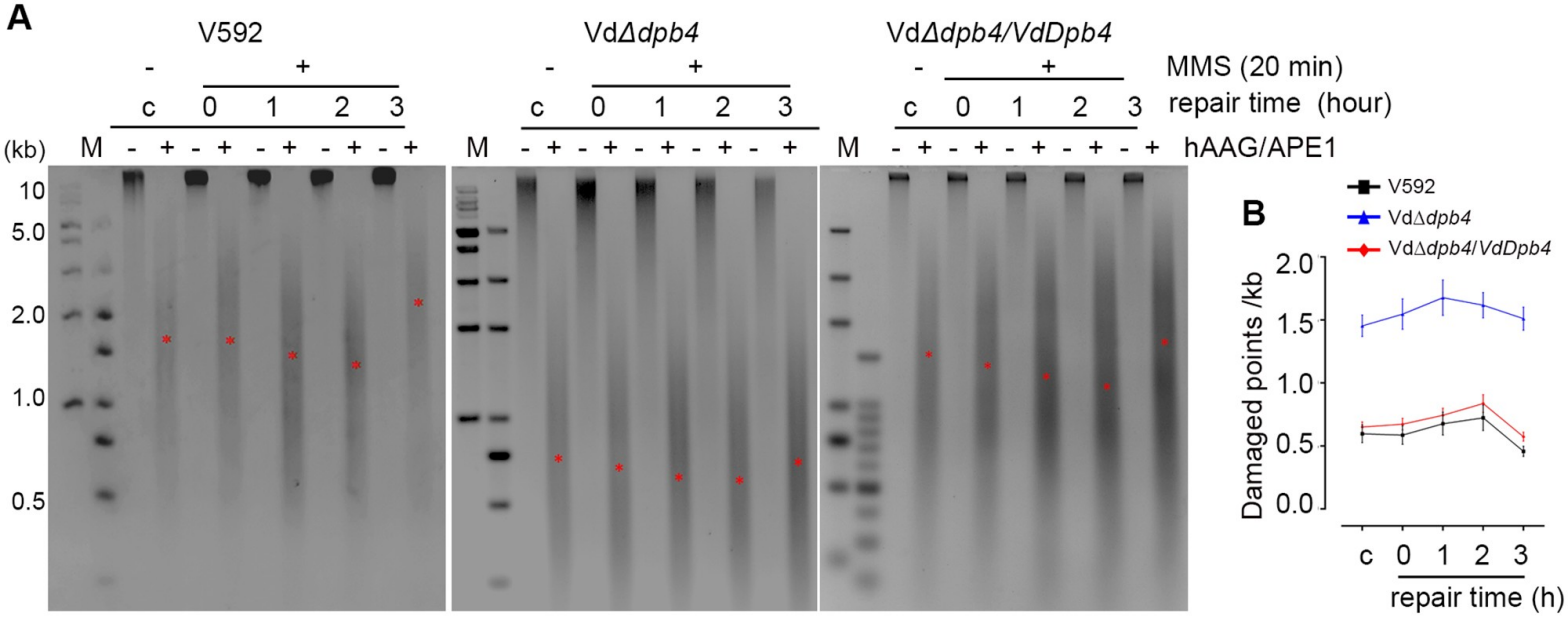
VdDpb4 is essential for resistance to DNA damage repair. A. The depletion of VdDpb4 dramatically inhibited genome-wide BER. A representative gel shows the repair time course in WT, the VdΔ*dpb4* mutant and complementation VdΔ*dpb4/VdDpb4* strains. M: marker DNA size standard; c: untreated cells; 0–3: cells damaged with MMS for 20 min; 1–3: repair time course in hours. Positions of the approximate median migration distance of the fragments in each lane are shown with red asterisks (*). B. The number of damaged DNA for each kilobase was calculated according to the average length obtained from the median migration distance. Error bars represent standard deviations from three independent experiments.

To verify the hypothesis that VdDpb4 is essential for DNA damage repair in *V*. *dahliae*, we examined the efficiency of alkylating-damaged triggered BER at the genome-wide level in the WT V592 and VdΔ*dpb4* mutant strains. The alkylating agent methyl methanesulfonate (MMS)-induced DNA damage is N-methylpurines (NMPs), which cause base mispairing and replication blocks [21] and are mainly repaired by BER in yeast [22]. We isolated the genomic DNA from WT V592, the VdΔ*dpb4* mutant and complementation VdΔ*dpb4/VdDpb4* strains that were exposed to MMS and collected at different repair time points, followed by analysis of the degree of DNA damage and repair efficiency [22]. As shown in Fig 3A, under normal culture conditions without MMS treatment, the number of damaged DNA per unit length in WT V592 and VdΔ*dpb4/VdDpb4* cells was much less than that in the VdΔ*dpb4* mutant (Fig 3A, lanes c+). With MMS treatment (without DNA repair), a higher frequency of damaged points in VdΔ*dpb4* cells than V592 and VdΔ*dpb4/VdDpb4* cells was observed, suggesting that VdΔ*dpb4* cells were more sensitive to MMS than V592 (Fig 3A, lanes 0+), consistent with the H_2_O_2_ treatment. In WT V592 and VdΔ*dpb4/VdDpb4*, the amount of MMS-damaged DNA slightly increased at early time points, but markedly decreased along with DNA repair at 3 hours (Fig 3A), indicating the efficient repair of NMPs during the repair time course. However, the frequency of repair of MMS-damaged DNA in VdΔ*dpb4* cells was much lower than that in V592 and VdΔ*dpb4/VdDpb4* cells, although the number of damaged DNA per unit length in the VdΔ*dpb4* mutant was reduced during the repair time course (Fig 3B). Taken together, we conclude that VdDpb4 is for essential DNA damage repair in *V*. *dahliae*.

### Identification of VdDpb4-associated proteins in vivo

**Fig 4:**
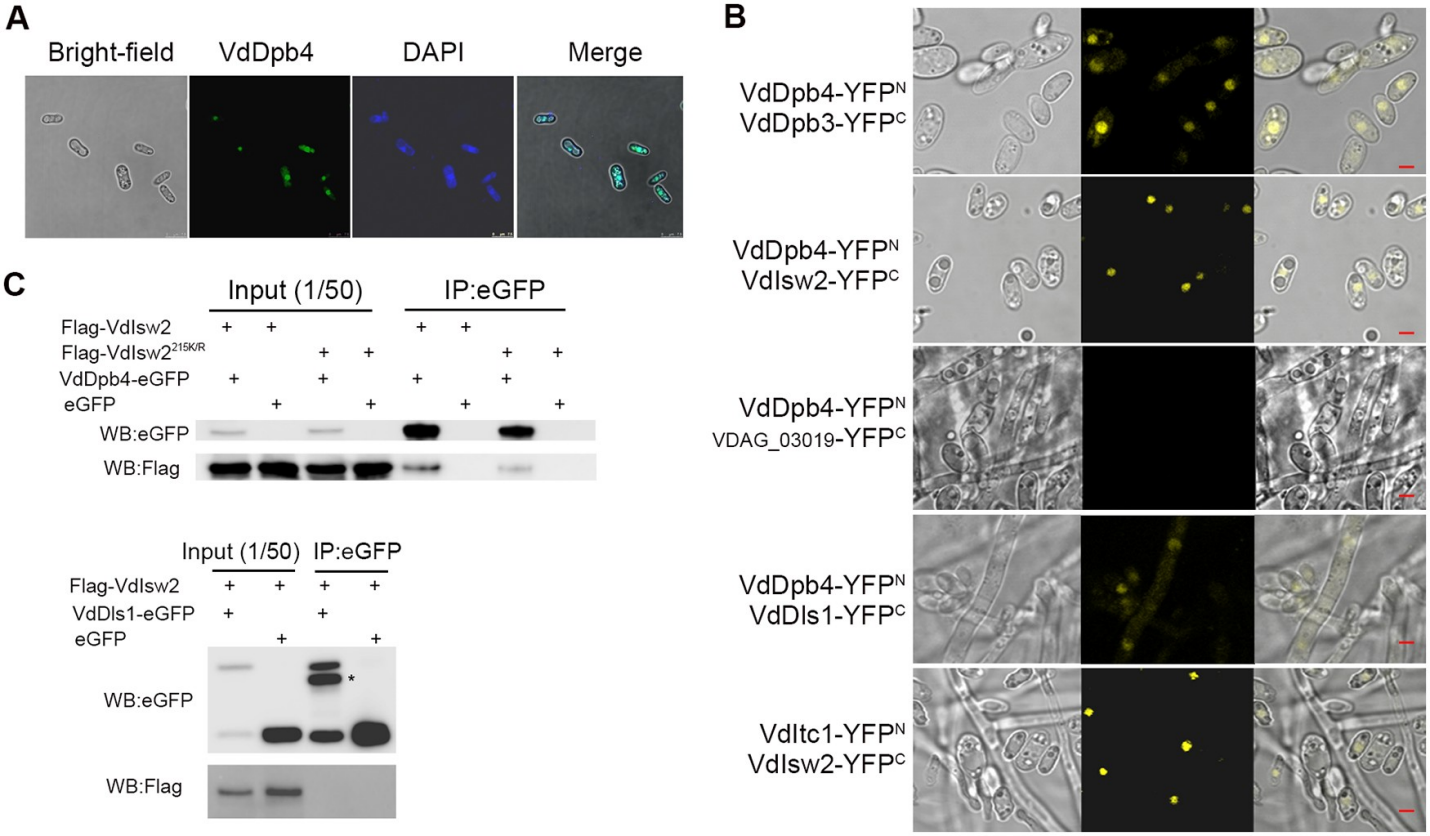
Identification of VdDpb4-associated proteins in vivo. A. Visualization of the location of VdDpb4 labeled with eGFP; conidia were stained with DAPI and then observed by fluorescence microscopy. Bar = 7.5 μm. B. BIFC assay showing the interaction of VdDpb4 and VdDpb3, and VdIsw2, and the interaction of VdIts1 and VdIsw2. All constructs were transformed into V592, and the signal was detected by fluorescence microscopy. VDAG_03019, a nuclear transcription factor Y subunit B-3 protein, was cotransformed with VdDpb4 as the negative control. Bar = 2.5 μm. C. Immunoblot of the coimmunoprecipitation. VdΔ*dpb4* expressing VdDpb4-eGFP and 3xFlag-VdIsw2, or Flag-VdIsw2^215K/R^ or eGFP, and V592 expressing Flag-VdIsw2 and VdDls1-eGFP, respectively, was cultured on MM medium for 3 days, and the conidia were collected for the Co-IP assay. *:a degraded protein or isoform.

To identify VdDpb4 interaction proteins in *V*. *dahliae*, eGFP-tagged VdDpb4 was expressed in VdΔ*dpb4* mutant to obtain VdΔ*dpb4*/*VdDpb4-eGFP* which exhibited WT virulence phenotype (S4A Fig). The VdDpb4-eGFP fusion protein was localized in the nucleus when observed under a fluorescence microscope (Fig 4A). We then pulled out GFP-tagged protein complexes from the fungal strain by using anti-GFP beads, followed by mass spectrometry (MS) analysis. The MS analysis identified peptides from nuclear proteins involved in several different pathways, such as DNA replication (e.g., VdPol2, VdDpb2), chromatin remodeling (e.g., VdIsw2 and VdItc1) and histone fold protein (e.g., VdDpb3) (S4B Fig and S2 Table).

For further verification, a bimolecular fluorescence complementation (BiFC) assay was performed. VdDpb4 was fused to the N-terminal fragment of YFP (YFP^N^), generating VdDpb4-YFP^N^, and the tested candidates VdDpb3 and VdIsw2, and the nuclear marker gene VDAG_03019 were each fused to the C-terminal fragment of YFP (YFP^C^) for transformation of the V592 strain. YFP signals were detected in transformants coexpressing VdDpb4-YFP^N^/VdDpb3-YFP^C^ or VdDpb4-YFP^N^/VdIsw2-YFP^C^ but not in the negative control expressing VdDpb4-YFP^N^/VDAG_03019-YFP^C^ (Fig 4B). The fourth component of ISW2, Dls1, was not obtained in MS analysis. The VdDls1 homologous sequence was searched from V592 based on a BLASTp search using yeast Dls1, and VdDls1-YFP^C^ was generated. Weak YFP signals were detected in transformants coexpressing VdDpb4-YFP^N^/VdDls1-YFP^C^ (Fig 4B), suggesting that ISW2 in *V*. *dahliae* also contained Dls1 homologs. Furthermore, the YFP signal was also detected in transformants coexpressing VdItc1-YFP^N^/VdIsw2-YFP^C^ (Fig 4B). We further verified the interaction of VdDpb4 and VdIsw2 by a coimmunoprecipitation (Co-IP) assay. A eGFP tag vector, a Flag-VdIsw2 and a Flag-VdIsw2^215K/R^ fusion construct, in which the lysine at the 215 site in the ATPase activity domain of VdIsw2 was substituted by a catalytically inactive arginine [23], were each introduced into the VdΔ*dpb4*/*VdDpb4-eGFP* strain. As shown in Fig 4C, Flag-VdIsw2, but not the vector control, coimmunoprecipitated with VdDpb4-eGFP, further confirming the in vivo interaction of the two proteins. Flag-VdIsw2^215K/R^ also coimmunoprecipitated with VdDpb4-eGFP, demonstrating that the ATPase activity is not required for VdIsw2-VdDpb4 interaction. No interaction between Flag-VdIsw2 and VdDls1-eGFP was detected in coimmunoprocipitation assay when they were coexpressed in fungal cells (Fig 4C). Therefore, we identified at least 4 chromatin-associated proteins, including the VdDpb4-VdIsw2-VdItc1 of chromatin remodeling ISW2 complex and VdDpb4-VdDpb3 of the DNA polymerase epsilon (Polε) complex.

The interaction between VdDpb4 and VdDpb3 is consistent with previous finding in yeast that Dpb4 and Dpb3 form heterodimers and cooperate with pol2 to form holo-Polε [24]. To determine whether VdDpb3 also participated in DNA damage repair pathways, we generated *VdDpb3* gene knockout mutants. No obvious different expression of *VdDpb4* in VdΔ*dpb3* mutants compared with in WT V592 (S4C Fig). Although Vd*Δdpb3* strains exhibited reduced pathogenicity and increased sensitivity to H_2_O_2_ (S4D and S4E Fig), the Vd*Δdpb3* mutants showed similar or slight increased resistance to MMS treatment compared to WT V592 (S4E Fig), suggesting that depletion of *VdDpb3* might release VdDpb4 from the Polε complex and enhance the effect of VdDpb4-containing ISW2 on DNA repair. We failed to obtain a mutant for either pol2 or VdDpb2, suggesting that deletion of the core component of Polε in *V*. *dahlia*e is fatal. Nevertheless, our data demonstrate that VdDpb4 localized in the nucleus and form a DNA Polε complex with VdDpb3 and chromatin remodeling ISW2 complex with VdItc1 and VdIsw2, and possibly VdDls1.

### VdIsw2 is required for development, DNA damage repair and pathogenicity

**Fig 5:**
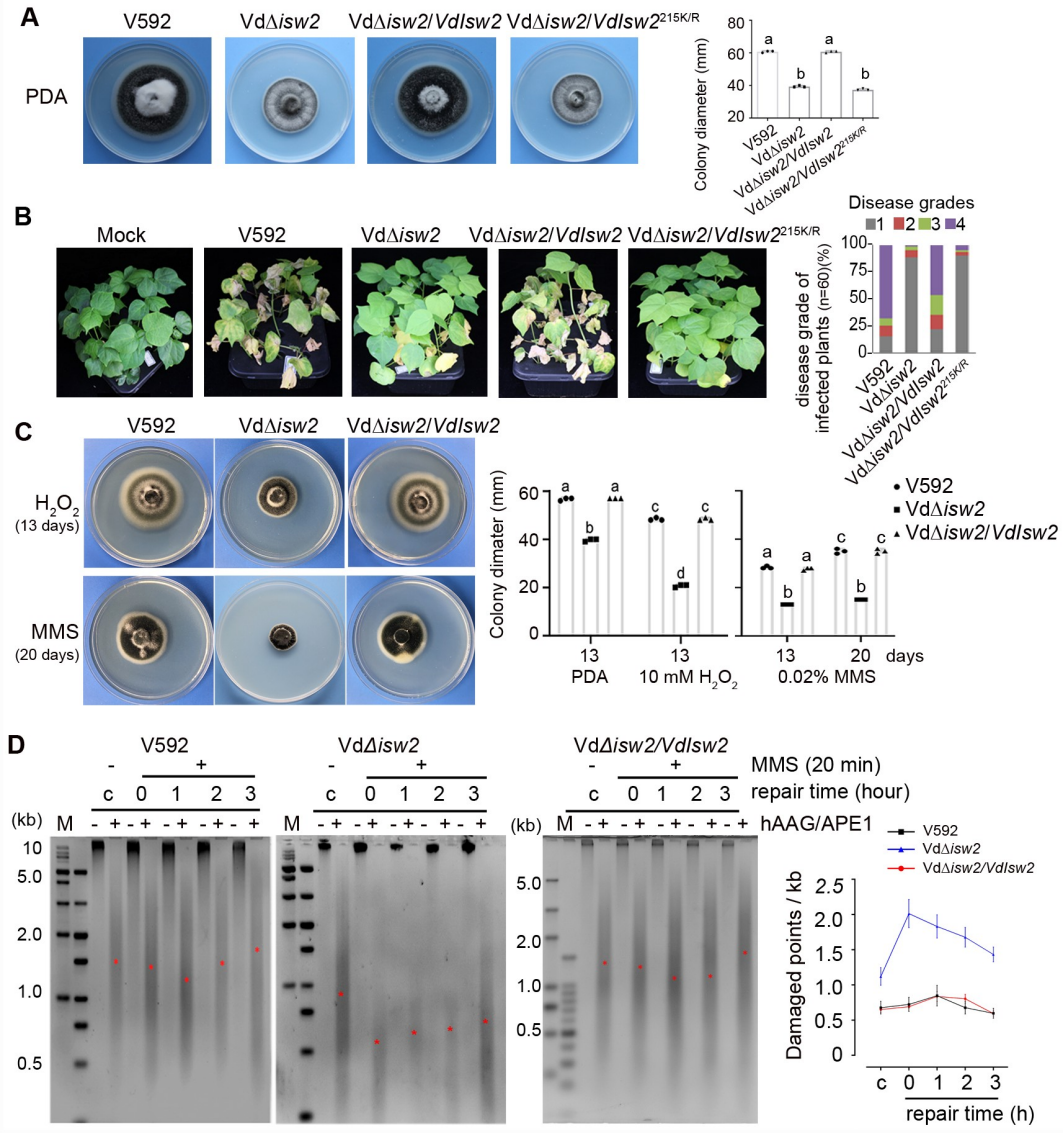
VdIsw2 is required for development, DNA damage repair and pathogenicity. A. Colony morphology of wild-type V592, the VdΔ*isw2* mutant strains, the VdΔ*isw2/VdIsw2* complementation strains, and the VdΔ*isw2/VdIsw2*^215K/R^ strains on PDA plates after 2 weeks postincubation. Quantification of colony diameter is shown on the right. Different letters indicate significant differences of fungal growth (*P* < 0.05, t-test, mean ± SD). B. Disease symptoms of cotton plants infected with V592, the VdΔ*isw2* mutant, VdΔ*isw2/VdIsw2*, and VdΔ*isw2/VdIsw2*^215K/R^ strains at 30 dpi. Disease grades were calculated as Fig 1. C. Quantification of colony diameter cultured on PDA media with H_2_O_2_ and MMS. Different letters indicate significant differences of fungal growth at *P*< 0.05, mean ± SD, one-way analysis of variance (ANOVA) followed by Tukey’s multiple comparisons test. D. The depletion of VdIsw2 dramatically inhibited genome-wide BER. A representative gel showing the repair time course in WT, the VdΔ*isw2* mutant and complementation VdΔ*isw2/VdIsw2* strains. M: marker DNA size standard; c: untreated cells; 0–3: cells damaged with MMS for 20 min; 1–3: repair time course in hours. Positions of the approximate median migration distance of the fragments in each lane are shown with red asterisks (*). The number of damaged DNA for each kilobase was calculated according to the average length obtained from the median migration distance. Error bars represent standard deviations from three independent experiments.

We next examined the VdDpb4-interacting protein VdIsw2 in pathogenicity and DNA damage repair. RT-qPCR analysis showed that the transcript level of *VdIsw2* was induced at an early infection time point during WT V592 infection in cotton and Arabidopsis Col-0 plants (S5A Fig). *VdIsw2* knockout mutant, Vd*Δisw2*, showed small colony morphology and exhibited markedly reduced virulence in cotton plants compared with WT V592 (Fig 5A and 5B). The defects in the grow morphology and pathogenicity of the Vd*Δisw2* mutant were restored in complementation strain Vd*Δisw2/VdIsw2*, but not in mutant strain Vd*Δisw2/VdIsw2*^215K/R^ (Fig 5A and 5B), demonstrating that the ATPase activity is required for VdIsw2 biological function, although it is not required for interaction with VdDpb4 (Fig 4C). We then detected the VdIsw2 function in DNA damage repair. As shown in Fig 5C, the Vd*Δisw2* mutant strain was sensitive to H_2_O_2_ and MMS compared with WT V592 and Vd*Δisw2/VdIsw2* strains (Fig 5C). Similar to the Vd*Δdpb4* mutant, the number of damaged DNA per unit length in Vd*Δisw2* mutant cells was much higher than that in WT V592 and Vd*Δisw2/VdIsw2* cells with or without MMS treatment (Fig 5D), and the frequency of repair of MMS-damaged DNA in VdΔ*isw2* cells was much lower than that in V592 and Vd*Δisw2/VdIsw2* cells during the repair time course (Fig 5D). Most genes involved in the DNA damage repair pathway were greatly decreased in the VdΔ*isw2* mutant compared to those in the V592 strain (S5B Fig). Consistently, the VdΔ*isw2* mutant also displayed reduced virulence on the Col-0 plants compared with WT V592 and complementation VdΔ*isw2/VdIsw2* strains (S5C Fig). The reduced virulence of the VdΔ*isw2* was partially impaired in the *rbohd* mutant plants (S5C Fig). Fungal biomass was significantly reduced in VdΔ*isw2*-infected Col-0 and *rbohd* plants compared with V592-infected ones (S5D Fig), suggesting that, in addition to reducing tolerance to RBOHD-produced ROS, other defects of VdΔ*isw2* mutant also impacted on its pathogenicity. Taken together, our data demonstrate that VdIsw2 plays roles in fungal development, DNA damage repair and virulence and reveal that the interplay between ISW2 chromatin remodelers and DNA repair regulates gene expression in response to DNA damage in *V*. *dahliae* and contributes an essential role in pathogenicity during plant infection.

### VdDpb4 is essential for chromatin structure maintenance and VdIsw2-dependent transcriptional regulation

**Fig 6:**
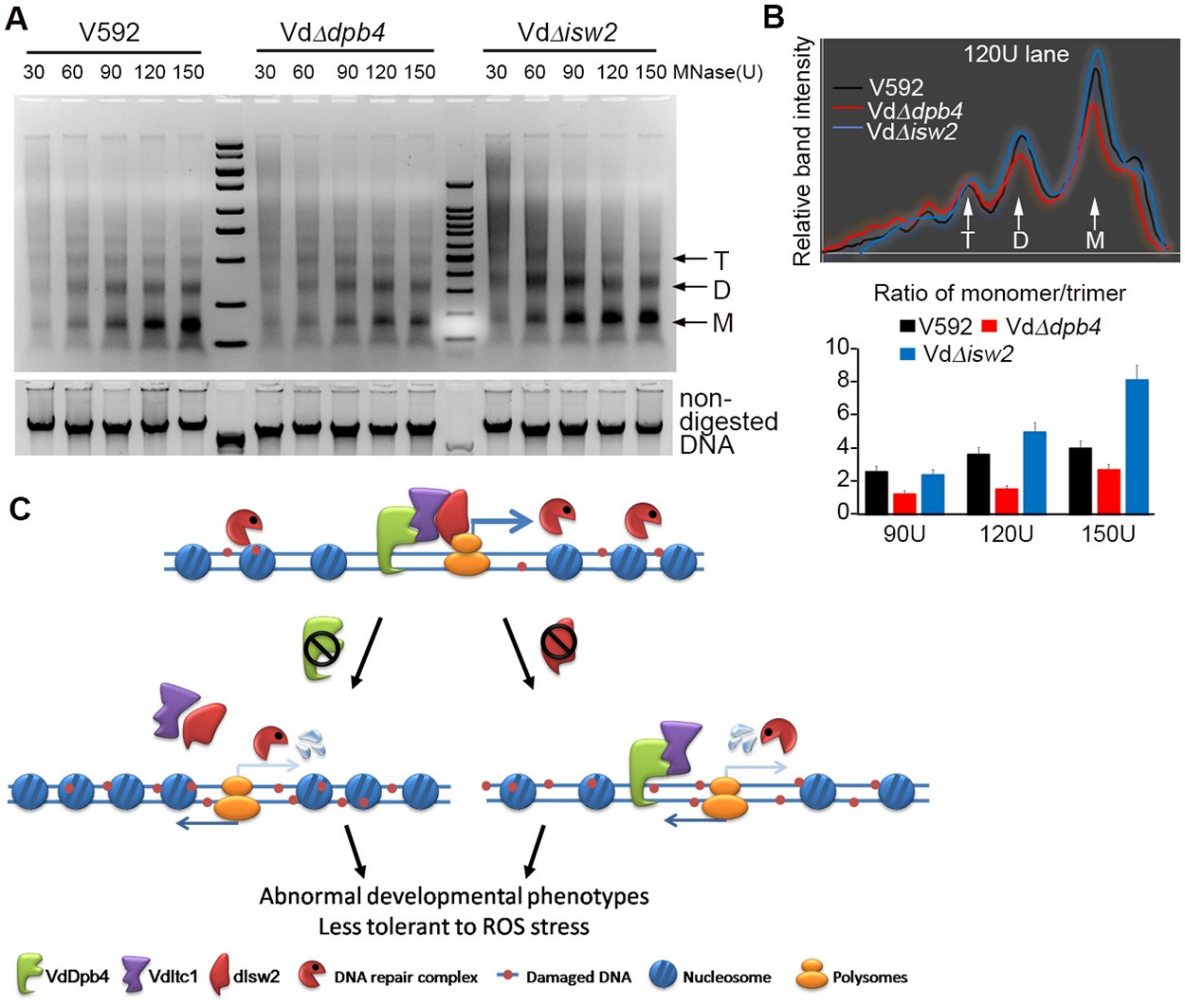
VdDpb4 is essential for chromatin structure maintenance. A. MNase digestion patterns in the wild-type V592, VdΔ*dpb4* and VdΔ*isw2* mutant cells synchronized at the G2/M phase of the cell cycle. The gel shows a representative tendency of three independent experiments with 20 min MNase digestion. M: DNA size standard, T: trinucleosome, D: dinucleosome, M: mononucleosome. B. Relative band intensity profiles of the 120 U MNase lanes for V592, VdΔ*dpb4* and VdΔ*isw2*. Quantitative analysis of MNase accessibility expressed as the ratio of mono- to trinucleosome signals at different concentrations of MNase is shown on the right. Error bars represent standard deviations. C. Proposed model for the VdDpb4- and VdIsw2-mediated complex control of the development and pathogenicity of *V*. *dahliae*. The interaction between VdDpb4 and VdIsw2 plays a role in maintaining normal chromatin structure, which is crucial for DNA repair and gene expression. However, depletion of VdDpb4 or VdIsw2 disrupts chromatin structure, which causes an imbalance in gene expression and impedes DNA damage repair and ultimately leads to an abnormal phenotype and less tolerance to ROS stress during development and plants infection.

The ISW2 chromatin remodeling complex has been shown to modulate chromatin structure locally and globally and has been implicated in nucleosome mobilization to improve DNA repair efficiency [25, 26]. To determine the possibility that disrupted gene expression and low-efficiency of DNA repair were linked with a change in chromatin structure in pathogenic fungal *V*. *dahliae*, we examined the accessibility of bulk chromatin by assessing Micrococal Nuclease (MNase) digestion patterns in WT V592, VdΔ*dpb4* and VdΔ*isw2* cells. All fungal cells were synchronized at the G2/M phase with nocodazole [22], and protoplasts were prepared. The chromatin was extracted by lysing the protoplasts and subsequently treated with increasing concentrations of MNase with different digestion times. The digested DNA was concentrated and then separated on agarose gels. As shown in Fig 6A and supplementation S6A Fig, in general, the VdΔ*dpb4* was resistant to MNase digestion, whereas VdΔ*isw2* was susceptible to MNase digestion compared with WT V592. To quantify these observations, band intensities corresponding to mono- and tri-nucleosomes were determined. Compared to WT V592, significantly lower ratio of mono- to trinucleosomes was obtained in VdΔ*dpb4* cells at each concentration of MNase treatment, indicating that the chromatin structure was more compacted and monomer nucleosomes were released more slowly from the chromatin; in contrast, in VdΔ*isw2* cells, the ratio was significantly increased, indicating that the chromatin structure was more flexible and monomer nucleosomes were released more quickly from the chromatin (Fig 6B). These results suggested that VdDpb4 may play an important role in maintaining a more “open” and accessible chromatin landscape (Fig 6C model), while VdIsw2 may play an antagonistic role against VdDpb4, and the genes were fine-tune regulated during development and infection due to the interaction of these two proteins.

Indeed, it has been shown that Isw2 functions adjacent to promoter regions where it repositions nucleosomes at the interface between genic and intergenic [27]. To determine whether VdIsw2 bound the promoter of genes involved in DNA damage repair, the Flag-VdIsw2 fusion construct was introduced into the Vd△*isw2* mutant, generating the VdΔ*isw2*/Flag-VdIsw2 strain, followed by performing a chromatin immunoprecipitation assay (ChIP). The ChIP results showed that VdIsw2 bound to the promoter of genes involved in DNA damage repair (S6B Fig). Notably, the enrichment of VdIsw2-bound DNA repair genes was decreased when the *VdDpb4* gene was deleted in the VdΔ*isw2*/Flag-VdIsw2 strain (S6C Fig). These data are in agreement with the above finding that VdDpb4 functions in maintaining a more “open” and accessible chromatin landscape for the location of ISW2 chromatin remodelers on DNA (Fig 6C). To further examine the VdIsw2-dependent transcriptional effect on gene expression, we investigated whether aberrant chromatin accessibility affected the recruitment of RNA polymerase II (pol II). ChIP coupled to qPCR was performed to analyze the occupancy of pol II in two VdIsw2-associated promoters in WT V592, VdΔ*dpb4* and the VdΔ*isw2* mutants. The ChIP-qPCR results showed that the recruitment of pol II was decreased in VdΔ*dpb4* cells compared to V592 (S6D Fig). Taken together, our data suggest that the aberrant chromatin accessibility caused by mutation of *VdDpb4* might affect the nucleosome spacing properties of the ISW2 complex and the subsequent recruitment of pol II to the DNA, leading to abnormalities in gene expression. Interestingly, the ChIP-qPCR results showed little difference in the recruitment of pol II to the tested DNA repair genes in the VdΔ*isw2* cells compared to V592 (S6E Fig). Because the expression of the two genes was significantly reduced in the VdΔ*isw2* mutants (S5B Fig), our data suggest that VdIsw2 is required for appropriate transcription instead of pol II binding to DNA. Nevertheless, our data suggest that VdDpb4 is required for the location of ISW2 on DNA and the VdIsw2-dependent transcriptional regulation of gene expression.

## Discussion

In the present study, through the identification of the fungal virulence gene *VdDpb4* from *V*. *dahliae*, we found that *VdDpb4* is essential for the *V*. *dahliae* development and infection, including tolerant toward ROS stress during development and plant infection. Further characterization of VdDpb4 and its interacting protein, VdIsw2, we show that the *V*. *dahliae* VdDpb4/VdIsw2-associated ISW2 complex plays essential roles in maintaining chromatin structure, regulating gene expression and promoting DNA repair. Several lines of evidence demonstrate that VdDpb4 functions as a ligand of the ISW2 chromatin remodeling complex for the regulation of gene expression during *V*. *dahliae* development and infection. (1) The localization of VdDpb4 and the interaction between VdDpb4 and VdIsw2 in the nucleus in V592 and the increased expression pattern of *VdDpb4* and *VdIsw2* during the early infection time points. (2) Knocking out *VdDpb4* affected the binding of VdIsw2 to gene promoters involved in DNA damage repair, and knocking out either *VdDpb4* or *VdIsw2* significantly reduced their expression. (3) Mutants with either *VdDpb4* or *VdIsw2* knocked out had significantly reduced virulence presumably due to the loss of resistance to DNA damaging agents, such as hydrogen peroxide, which is produced by plants to protect themselves from infection. (4) Both VdDpb4 and VdIsw2 are required for maintaining normal chromatin structure. Depletion of *VdDpb4* made the chromatin structure more compact, while elimination of *VdIsw2* caused the opposite result, and either the depletion of *VdDpb4* or elimination of *VdIsw2* affected the efficiency of DNA repair, at least by the BER repair pathway.

### VdDpb4 plays an important role in the interplay between chromatin remodelers and DNA repair in *V*. *dahliae*

The four known families of chromatin remodeling complexes, the SWI/SNF family, ISWI family, CHD family, and INO80 family, share a similar ATPase domain and have the ability to alter histone-DNA interactions [7]. We also found that the ATPase activity is required for VdIsw2 biological functions, albeit not for the interaction with VdDpb4. The yeast yISW2 complex, consisting of the four subunits: Dpb4, Dls1, Isw2 and Itc1 [24, 28], resembles the smaller subunits of the CHRAC (Chromatin Accessibility Complex) complex found in *Drosophila* and humans [29, 30]. Depletion of the *Drosophila* histone-fold protein CHRAC14 and human CHRAC17, which are homologous genes of VdDpb4, also reduced the efficiency of DNA repair [29, 30]. In yeast, the binding of Dpb4 to Isw2 and Itc2 was found to require Dls1 [31]. We observed a weak interaction between VdDpb4 and VdDls1 by BiFC assay. However, VdDls1 neither interacted with VdIsw2, nor being detected in the VdDpb4-mediated mass spectrometry analysis in which VdIsw2 and VdItc1 were readily detected. Considering the fact that knocking out *VdDpb4* alone resulted in a reduction in VdIsw2 binding, as well as Pol II recruitment, to the promoter of genes involved in DNA damage repair, our data suggest that VdDls1 might not be required for the binding of VdDpb4 to other components of the ISW2 complex in *V*. *dahliae*. We found that VdDpb4 and VdIsw2 function in chromatin organization and transcription. Depletion of *VdIsw2* did not affect Pol II recruitment to DNA but did affect its appropriate transcription, which was in agreement with the finding that *Saccharomyces cerevisiae* Isw2 repositions nucleosomes to enforce transcription by preventing transcription initiation from cryptic sites [27]. Because depletion of *VdDpb4* resulted in a more compacted chromatin structure, our data demonstrate that VdDpb4 is required for maintaining a more “open” and accessible chromatin landscape, serving as a ligand for the localization of ISW2 on DNA to balance the nucleosome spacing properties with VdIsw2, as well as for the VdIsw2-dependent transcriptional effect on gene expression, especially in response to DNA damage caused by plant-produced defensive ROS during plant infection.

### VdDpb4-containing DNA polymerase epsilon plays a role in the development and pathogenicity of *V*. *dahliae*

In mammalian cells, DNA polymerase epsilon (Polε) is associated with a multiprotein complex that contains most of the essential components of the BER pathway, including APE1, XRCC1, and DNA ligase1, to perform the preassembled DNA repair machinery, which is recruited to base damage and breaks occurring at replication forks [32]. In addition to chromatin remodeling of the ISW2 complex, we also identified VdDpb4 in the Polε complex associated with VdDpb3. In *Schizosaccharomyces pombe*, Dpb4 and Dpb3 were reported as the two smallest subunits of Polε [33]. In *Saccharomyces cerevisiae*, ScDpb4 was also identified as a component of Polε and played a significant role in maintaining the complex structure of polε [34]. We failed to obtain a mutant for either the core component of Polε, pol2 or VdDpb2, suggesting that the chromatin-associated Polε complex is pivotal for *V*. *dahliae* developmental growth. In *S*. *pombe*, depletion of Dpb3 led to an accumulation of cells in the S phase that exhibited an abnormal phenotype with increased cell length [33]. Interestingly, we found that with depletion of *VdDpb4*, but not *VdDpb3*, the fungus developed longer conidiospores with a reduced hyphal growth rate. Unlike VdDpb4, VdDpb3 did not play a role in DNA damage repair in *V*. *dahliae*. However, depletion of either *VdDpb4* or *VdDpb3* increased the sensitivity to H_2_O_2_, and remarkably reduced virulence in cotton plants. Our data add novel virulence functions of the Polε complex in pathogenic fungi, in addition to its role in fungal development.

### Regulatory mode of VdDpb4 and VdIsw2 in *V*. *dahliae* during plant infection

During plant infection, the expression levels of *VdDpb4* and *VdIsw2* increased at early time points. However, while *VdIsw2* expression returned to the basal level at a later time point, the expression level of *VdDpb4* constantly increased. VdDpb4 participating in the ISW2 complex and Polε complex may account for its constant expression pattern during the plant infection process. *V*. *dahliae* is a hemibiotrophic fungus that grows preferably in vascular tissues of host plants from the parasitic phase to the saprophytic phase, when conidia and microsclerotium are produced [35, 36]. We previously found that upon inoculation, conidia had germinated and extended hyphae on the root surface, only a few of which penetrated intercellularly into the epidermal cells by 48 hours postinoculation (hpi) and had hyphal growth within the root cortex in a directed manner toward the central cylinder. Upon successfully reaching the xylem vessel cells, internal hyphal ramification began by 72 hpi, and the fungal biomass increased over time [36]. The increase in *VdDpb4* and *VdIsw2* at early time points coincides with the requirement for the fungal resistance to DNA damage caused by plant-produced defensive ROS for successful penetration. However, only VdDpb4 is constantly required for fungal ramification and conidia formation within the xylem vessel cells at later infection time points. The reduction in and maintenance of the basal level of *VdIsw2* expression at later time points may provide more and/or active Polε complex required for the fungus growth inside the plant vasculature tissue. Our findings reveal that as a plant pathogenic fungus, chromatin-associated proteins not only play roles in the interplay between chromatin remodeling and DNA repair for development and initial infection upon ROS stress, but also extend their regulatory mode to development and virulence processes during plant infection against various defensive signaling.

VdDpb4 homologs were highly evolutionarily conserved in phytopathogenic fungi. Although more components and functions of ISW2 and Polε complexes in *V*. *dahliae* remain to be exploited, our findings suggest that VdIsw2 and VdDpb4 chromatin remodelers are implicated in global chromosome structure, the regulation of transcription and DNA repair in *V*. *dahliae*. Our data demonstrate that the timely changes in chromatin structure to regulate related physiological processes play an essential role in pathogenic fungi in response to stresses during development and plant infection and provide the first example and essential information for further investigation of chromatin-associated complexes in pathogenic fungi.

## Materials and methods

### Fungal isolates, culture conditions and infection assays

The virulent defoliating *V*. *dahliae* isolate V592 from cotton that originated in Xinjiang, China, was used in this study. This isolate was stored at -80 °C and was recovered on potato dextrose agar (PDA) medium at 26 °C in the dark. The conidia for the infection assays were cultured in liquid Czapek-Dox medium (NaNO_3_ at 2 g/liter, K_2_HPO_4_ at 1 g/liter, KCl at 0.5 g/liter, MgSO_4_ at 0.5 g/liter, FeSO_4_ at 0.01 g/liter, sucrose at 30 g/liter) for 3 days.

For plant infection, cotton plants (`Xinluzao No. 16’) were used in infection assays to evaluate the effect of the *V*. *dahliae* isolate V592 and transformants on virulence using our laboratory’s unimpaired root-dip inoculation method, as described in our previous research [11]. For Arabidopsis infection assay, approximate two weeks seedlings of wild-type and *rbohd* mutant were transferred to soil that was mixed with 1×10^6^ cfu/ml spores of V592, Vd*Δdpb4*, Vd*Δdpb4*/*VdDpb4*, Vd*Δisw2*, Vd*Δisw2/VdIsw2*, respectively. Disease progression was recorded after approximate 3 weeks of incubation. The infection assay for the transformants was repeated three times. The symptoms were evaluated, and the disease grade was classified as follows: 1 (0–25% wilted leaves), 2 (25–50%), 3 (50–75%) and 4 (75–100%) [37].

### DNA and RNA extraction and cDNA

Nucleic acid extraction and fungal transformation methods have all been previously described [11]. Total RNA was isolated from frozen mycelium collected from Czapek-Dox medium after 3 days of cultivation. *V*. *dahliae* cDNA was reverse-transcribed using SuperScript1III (Invitrogen).

### Quantitative real-time PCR

Before reverse transcription, residual DNA was removed from the total RNA using gDNA wiper (Vazyme). cDNA was reverse transcribed using HiScript II Q RT Supermix (Vazyme), and qRT-PCR was performed using ChamQ SYBR qPCR MasterMix (Vazyme) with the Bio-Rad CFX96 Real-Time system. The transcription levels of the target genes were quantified relative to the constitutively expressed elongation factor 1-α of *V*. *dahliae* (VdElf). The gene-specific primers are listed in S1 Table. Biological replicates were performed three times.

### Penetration assays

For the penetration assays, sterilized cellophane membrane (DINGGUO, Beijing, China) was overlaid onto minimal medium (glucose at 2 g/liter, NaNO_3_ at 2 g/liter, KH_2_PO_4_ at 1 g/liter, MgSO_4_-7H_2_O at 0.5 g/liter, KCl at 0.5 g/liter, citrate at 10 mg/liter, ZnSO_4_-7H_2_O at 10 mg/liter, FeSO_4_-7H_2_O at 10 mg/liter, NH_4_Fe(SO_4_)_2_-12H_2_O at 2.6 mg/liter, CuSO_4_-5H_2_O at 0.5 mg/liter, MnSO_4_-H_2_O at 0.1 mg/liter, H_3_BO_3_ at 0.1 mg/liter, Na_2_MoO_4_-2H_2_O at 0.1 mg/liter and agar at 20 g/liter). The cultures were incubated on the cellophane membrane for 3 days. The membranes were removed, and hyphae were observed in the underlying medium to determine if there were any breaches in the cellophane.

### Stress treatment

For osmotic stress, fungi were cultured on PDA with 0.5 M NaCl or 0.6 M Sorbitol; for hydrogen peroxide stress, fungi were cultured on PDA with 10 mM H_2_O_2_; for DNA damage agent treatment, fungi were culture on PDA with 20 μg/ml Mitomycin C or 0.02%(v/v) MMS.

### Fungal biomass measure in planta

Tissues of infected Arabidopsis plants were harvested at approximate 3 weeks after incubation and flash-frozen in liquid nitrogen for DNA extraction. Fungal biomass in infected Arabidopsis plants was estimated by the value of the fungal-specific DNA relative to plant-specific DNA using quantitative real-time PCR.

### Construction and transformation

To generate the knockout plasmids pGKO-HPT-VdDpb3 and pGKO-HPT-VdIsw2, upstream and downstream genomic sequences were amplified with the following primer pairs: VdDpb3up-s/a and VdDpb3dn-s/a; and VdIsw2up-s/a and VdIsw2dn-s/a (S1 Table). Both sequences were inserted into a position flanking the hygromycin-resistant cassette of the vector pGKO with the USER enzyme to generate knockout plasmids. To generate the double knockout mutant, the knockout plasmid pGKO-NAT-VdDPB4 was constructed by replacement of the hygromycin-resistant cassette with a nourseothricin-resistant cassette, and transformation was performed as described previously [11].

To generate the complementary plasmid of pNEO-VdDpb4com, the wild-type VdDPB4 gene was amplified from the V592 genome with the primer pairs of VdDPB4 genomic-s/a (S2 Table). The VdDPB4 fragment (with stop codon or without) was inserted into the HindIII/EcoRI-linearized pSUL-NEO-Tef-EKGFP-TrpC binary vector, which contained a C-end eGFP [15]. The pNEO-VdDPB4com plasmid was transformed into the knockout mutants of VdΔ*dpb4* to produce complemented strains, VdΔ*dpb4/VdDpb4*. Transformants were selected on PDA medium with G418 at 40 mg/liter.

To generate the complementary plasmid of pNEO-VdIsw2com, the wild-type VdISW2 gene was amplified from the V592 genome with the primer pairs of VdISW2 genomic-s/a (S2 Table). The VdISW2 fragment was inserted into the BamHI/EcoRI-linearized pSUL-NEO-Tef-Flag-TrpC binary vector, which was modified by replacement of the eGFP tag with a Flag tag. The pNEO-VdISW2com plasmid was transformed into the knockout mutant VdΔ*isw2* to produce complemented strain, VdΔ*isw2/VdIsw2*. Transformants were selected on PDA medium with G418 at 40 mg/liter.

To generate the pSulPH-Neo-VNbackbone vector for the BiFC assays, the N- fragments of Venus, the G418 resistant cassette, the Tef promoter and the TrpC terminator were amplified with the primers of VN-s/a,G418BOX-s/a, Tef promoter-s/a, TrpC terminator-s/a, respectively. All the fragments were ligated into HindIII/XhoI-digested pSulPH based on homologous recombination cloning. (ClonExpressMultiS One Step Cloning Kit, Vazyme, China).

To generate the pSulPH-HPT-VC backbone vector for the BiFC assays, the C- fragments of Venus, the Hygromycin-resistant cassette, the oliC promoter and the TrpC terminator were amplified with the primers of VC-s/a, HPTBOX-s/a, oliC promoter-s/a, TrpC terminator-s/a, respectively. All the fragments were ligated into HindIII/XhoI-digested pSulPH based on homologous recombination cloning (ClonExpressMultiS One Step Cloning Kit, Vazyme, China).

To create the VdDpb4-YFP^N^ and VdItc1-YFP^N^ vectors, the cDNA of VdDpb4 and VdItc1 were amplified with the primers VdDPB4-cDNA-VN-s/a and VdIsw2-cDNA-VN-s/a, respectively, and the fragment was ligated into BamHI-digested pSulPH-Neo-VN.

To create the VdDpb3-YFP^C^, VdIsw2-YFP^C^ and VDAG_03019-YFP^C^, the cDNA of VdDpb3, VdIsw2 and VDAG_03019 were amplified with the primer VdDpb3-cDNA-VN-s/a, VdIsw2-cDNA-VN-s/a and VDAG_03019cDNA-VN-s/a, respectively, and the fragment was ligated into BamHI-digested pSulPH-HPT-VC.

VdDpb4-YFP^N^/VdDpb3-YFP^C^, VdDpb4-YFP^N^/VdIsw2-YFP^C^, VdItc1-YFP^N^/VdIsw2-YFP^C^ and VdDpb4-YFP^N^/VDAG_03019-YFP^C^ were cotransformed into V592, and transformants with G418 and hygromycin resistance were selected.

### Interaction assays

For the BiFC assay, the transformants with VdDpb4-YFP^N^/VdDpb3-YFP^C^, VdDpb4-YFP^N^/VdIsw2-YFP^C^, VdItc1-YFP^N^/VdIsw2-YFP^C^ and VdDpb4-YFP^N^/VDAG_03019-YFP^C^ were inoculated on PDA medium for 3 days. Then, fungal colonies were removed for fluorescence microscopy observation. For the co-IP assay, VdΔ*dpb4* expressing VdDpb4-eGFP and 3xFlag-VdIsw2, or 3xFlag-VdIsw2^215K/R^ or eGFP-vector, and VdΔ*isw2* expressing Flag-VdIsw2 and VdDls1-eGFP or eGFP, respectively, were cultured in Czapek-Dox medium for 3 days. Then, conidia and mycelium were harvested, and protein was extracted with liquid nitrogen and lysis buffer (20 mM Tris-HCL pH 8, 150 mM NaCl, 1% Triton X-100 and 1 x Protease inhibitor (Roche)). Total protein extract was incubated with eGFP-beads (2-4090-002, IBA Life science) or Flag M2 beads (Sigma) overnight at 4 °C. Protein detection was conducted with the Flag antibody (1:1000 dilution, Sigma) or eGFP antibody (BE2076-100 μL; 1:5000 diluted, EASY-BIO).

### ChIP assay

ChIP was performed as described [38]. Briefly, all the strains were were cultured in liquid Czapek-Dox medium for 3 days and then were fixed with 1% formal dehyde for 15 min. Approximate 0.5 g fresh weigh conidia and mycelium were used for the chromatin immuneprecipitation assay. For IP, the corresponding antibody was used to pull down chromatin containing the specific target. Then, qPCR was performed to determine the enrichment of immunoprecipitated DNA in the ChIP assay.

### DNA repair assay

V592, VdDpb4 and theVdIsw2 mutant strains were first cultured in Czapek-Dox medium for 3 days. Second, all cultures were synchronized at the G2/M phase of the cell cycle with the addition of nocodazole (15 μg/ml) and grown for an additional 6 hours: subsequently, 0.2% MMS (final concentration) was added to the cultures, and the cultures were incubated in a shaker for 20 minutes at 26 °C. Third, cells were harvested and washed with ddH_2_O and resuspended in ddH_2_O and allowed to repair at 26 °C for varying time points. Aliquots for the damage-repair time course were collected, and genomic DNA was extracted. Fourth, genomic DNA was digested with hAAG glycosylase and APE1 endonuclease for 6 hours at 37 °C,resolved on alkaline agarose gels and stained with SYBR gold (Invitrogen). To examine global BER, the number-average length of genomic DNA (±AAG/APE treatment) was determined and used to calculate the average number of ssDNA/kb, as described previously [39].

### MNase assay

V592, VdDpb4 and VdIsw2 mutant strains were first cultured in Czapek-Dox medium for 3 days. Second, all cultures were synchronized at the G2/M phase of the cell cycle with the addition of nocodazole (15 μg/ml) and grown for an additional 6 hours. Third, the conidia and mycelium were harvested by centrifugation and resuspended with TE (10 mM Tris-HCl, 1 mM EDTA pH8.0, 1.4ml/g). The final volume was brought up to 3.5 ml/g with water,β-mercaptoethanol was added to a final volume ratio of 1/200, and the stains were incubated in a shaker for 30 minutes at 26 °C. Fourth, all the strains were harvested and washed with 1.2 M KCl for twice and resuspensed in lysis buffer for digestion of the fungal cell wall for 3 hours, and the strains were checked for the presence of spheroplasts several times during the digestion. The spheroplasts were washed with 1.2 M KCl to remove the residual enzyme. Fifth, one-tenth of the spheroplasts were frozen in liquid nitrogen for DNA extraction as non-digested control. The rest spheroplasts (~1x10^8^) were resuspended in digestion buffer (10 mM Tris-HCl, PH7.4, 50 mM NaCl, 5 mM MgCl_2_, 1 mM CaCl_2_, 1 mM β-mercaptoetanol, 1% TritonX-100, 0.1% sodium deoxycholate, and PMSF), divided into five equal parts and digested with varying concentrations of MNase (Thermo Fisher Scientific) at 37 °C for 15 or 20 min. Reactions were stopped with 5% SDS and 50 mM EGTA, and treated with proteinase K for 2 hours at 55 °C. Finally, digested DNA was purified and resolved on 1.2% native agarose gels.

### Measurements of SOD enzyme activity

V592 and VdΔ*dpb4* mutant strain were first cultured in Czapek-Dox medium for 3 days then harvested for SOD enzyme activity measurement. SOD activity was determined using a SOD assay Kit (BC0170, Solarbio, China) based on the xanthinoxidase method [40].

### Statistical analysis

The fungal growth experiments were performed at least 3 times. The RT-qPCR was performed at least 3 times. The data were exhibited as mean + SD. Student’s test was utilized to evaluate data between 2 groups, while the comparison among multiple groups was conducted by one-way ANOVA. Statistical analysis was performed with GraphPad Prism 6.0 software. Significance level was set as *P* < 0.05.

## Supporting information

S1 Fig Phylogenic analysis of the VdDbp4 homologs with other Dpb4 proteins. A. Schematic representation of the VdDpb4 protein comparison of the histone-fold motif and coiled-coil domain. Phylogenic analysis of the VdDbp4 homologs with other Dpb4 proteins available from a limited number of fungal species as indicated. VdDbp4 is labeled. The analysis was performed using the neighbor-joining method phylogeny test with the bootstrap method (No. of bootstrap replications = 1000). B. Schematic representation of the homologous recombination event involved in the targeted replacement of fungal gene. C. Southern blot analysis of targeted gene deletion mutants. Hind III digested genomic DNA from V592 wild type strain and two putative of VdΔdpb4, VdΔisw2, and VdΔdpb3 transformants were blotted with the probe indicated on the top of gels. D. Reverse transcription qPCR (RT-qPCR) analysis of the expression of VdDpb4, VdIsw2 and VdDpb3 in knockout mutants with primers listed in S1 Table.
(TIF)

S2 Fig Penetration assay with a cellophane membrane. A. Penetration assay with a cellophane membrane. Colonies of V592, the VdΔdpb4 mutant strains, and the VdΔdpb4/VdDpb4 complementation strains grown on MM medium overlaid with a cellophane layer (above) and removal of the cellophane membrane (below). Photographs in the first row were taken at 3 dpi. The second row shows growth of V592, the VdΔdpb4 mutant strains, and the VdΔdpb4/VdDpb4 complementation strains on MM medium after penetration of the cellophane membrane. B. Statistical analysis of the hyphopodia on the cellophane membrane at 3 dpi. Differentiation of hyphopodia (swollen hyphae) in V592 and VdΔdpb4 is indicated by arrows. More than three areas were counted by random selection, and the average number of hyphopodia was calculated. Error bars represent standard deviations. Hyphopodium could penetrate the cellophane membrane and grow under the membrane as showed and indicated with arrows. Asterisks indicate significant differences (P < 0.05, t-test, mean ± SD). C. VdDpb4 expression was rapidly induced at early time points during cotton infection as detected by RT-qPCR). Different letters indicate significant differences of gene expression at P< 0.05, mean ± SD, one-way analysis of variance (ANOVA) followed by Tukey’s multiple comparisons test.
(TIF)

S3 Fig VdΔdpb4 mutation significantly reduced gene expression involved in DNA damage repair. A. Mycelial growth on PDA agar medium with NaCl and sorbitol and quantification of colony diameter. B. RT-qPCR analysis of the expression level of the catalase-encoding genes in the V592 and VdΔdpb4 mutant strains. Error bars represent standard deviations. C. RT-qPCR analysis of the expression level of the SOD-encoding genes in the V592 and VdΔdpb4 mutant strains. Asterisks indicate significant differences (P<0.05; t-test, mean ± SD). D. RT-qPCR analysis of the expression level of the genes involved in DNA damage repair. Asterisks indicate significant differences (P<0.05; t-test, mean ± SD). (for B-D, the name description and function of the genes analyzed were listed below).
(TIF)

S4 Fig Identification of VdDpb4-associated proteins in V. dahliae, and detection of Vd△dpb3 mutant strains in pathogenicity and stress response. A. VdDpb4-eGFP expression in VdΔdpb4 mutant restored virulence of the mutant in cotton plants. B. The proteins identified by mass spectrometry analysis of purified VdDpb4 were grouped on the basis of their functions. Full list of proteins identified is shown in S2 Table. C. Expression of VdDpb4 in V592 and VdΔdpb3 mutants. (ns: no significant difference, t-test, mean ± SD). D. Disease symptoms of cotton plants infected with V592 or VdΔdpb3 at 30dpi. Disease grades on cotton leaves were classified into four levels dependent on the ratio of (wilted and dropped off leaves / total leaves) during fungal invasion: grade 1 = 0–25%; grade 2 = 26–50%; grade 3 = 51–75%; and grade 4 = 76–100%. E. Quantification of colony diameter cultured on PDA media with H2O2 and MMS. Different letters indicate significant differences of fungal growth at P< 0.05, mean ± SD, one-way analysis of variance (ANOVA) followed by Tukey’s multiple comparisons test).
(TIF)

S5 Fig VdIsw2 plays an essential role in responding to RBOHD-mediated defenses during infection. A. VdIsw2 expression was induced at early time points during cotton and Arabidopsis plant infection as detected by quantitative RT-PCR (RT-qPCR). Different letters indicate significant differences of gene expression at P< 0.05, mean ± SD, one-way analysis of variance (ANOVA) followed by Tukey’s multiple comparisons test). B. RT-qPCR analysis of the expression level of genes involved in DNA damage repair (gene names were shown in S3 Fig). Asterisks indicate significant differences, ns: no significant difference, (P<0.05; t-test, mean ± SD). C. VdIsw2 is essential for resistance to RBOHD-mediated defense. Disease symptoms of wild-type (Col-0) and rbohd mutant Arabidopsis plants infected with V592, mutant or complementation strains at 20 dpi. The disease grades were evaluated with three replicates of 48 plants for each inoculum. D. Reduced fungal biomass in VdΔisw2-infected Arabidopsis plants compared with V592-infected ones at 2-week postinoculation. The values were quantitative real time (qPCR) of fungal tubulin DNA relative to Arabidopsis At4g33380 DNA. Statistical analysis was described as in A.
(TIF)

S6 Fig VdIsw2 plays an essential role for chromatin structure maintenance and regulating gene expression involved in DNA damage repair during infection. A. MNase digestion patterns in the wild-type V592, VdΔdpb4 and VdΔisw2 mutant cells synchronized at the G2/M phase of the cell cycle. The gel shows an experiment with 15 min MNase digestion. M: DNA size standard, T: trinucleosome, D: dinucleosome, M: mononucleosome. B. ChIP-qPCR analysis showing that VdIsw2 could bind to the gene promoter region involved in DNA damage repair. ChIP assays were conducted in cells expressing Flag-VdIsw2 using an anti-Flag antibody. C. ChIP-qPCR analysis showing that VdIsw2 binding to the gene promoter region was reduced in the Flag-VdIsw2/VdΔdpb4 mutant strain compared with the wild-type Flag-VdIsw2 strain. ChIP assays were conducted in cells expressing Flag-VdIsw2 using an anti-Flag antibody. Tubulin was used as a control. D. ChIP-qPCR analysis showing that the recruitment of RNA polymerase II was reduced in the VdΔdpb4 mutant strain compared with the wild-type V592 strain. ChIP assays were conducted with an anti-Pol II antibody. Tubulin was used as a control. E. ChIP-qPCR analysis showing little difference in the recruitment of pol II to the tested DNA repair genes in the VdΔisw2 mutant strain compared with the wild-type V592 strain. Tubulin was used as a control. (For B-E, gene name and description were in S3 Fig. For D-E, a scheme at right shows where the oligonucleotides used in each gene.
(TIF)

S1 Table Primer list in this study. (PDF)

S2 Table Full list protein identified by mass spectrometry analysis. (XLSX)

## References

[1] Kadota Y, Shirasu K, Zipfel C. Regulation of the NADPH Oxidase RBOHD During Plant Immunity. Plant Cell Physiol. 2015;56(8):1472–80. Epub 2015/05/06. 10.1093/pcp/pcv063 . 25941234

[2] Kadota Y, Sklenar J, Derbyshire P, Stransfeld L, Asai S, Ntoukakis V, et al Direct regulation of the NADPH oxidase RBOHD by the PRR-associated kinase BIK1 during plant immunity. Mol Cell. 2014;54(1):43–55. Epub 2014/03/19. 10.1016/j.molcel.2014.02.021 . 24630626

[3] Li L, Li M, Yu L, Zhou Z, Liang X, Liu Z, et al The FLS2-associated kinase BIK1 directly phosphorylates the NADPH oxidase RbohD to control plant immunity. Cell Host Microbe. 2014;15(3):329–38. Epub 2014/03/19. 10.1016/j.chom.2014.02.009 . 24629339

[4] Lindahl T. Suppression of spontaneous mutagenesis in human cells by DNA base excision-repair. Mutat Res. 2000;462(2–3):129–35. Epub 2000/04/18. 10.1016/s1383-5742(00)00024-7 . 10767624

[5] Ataian Y, Krebs JE. Five repair pathways in one context: chromatin modification during DNA repair. Biochem Cell Biol. 2006;84(4):490–504. Epub 2006/08/29. 10.1139/o06-075 . 16936822

[6] Yadon AN, Tsukiyama T. SnapShot: Chromatin remodeling: ISWI. Cell. 2011;144(3):453–e1. Epub 2011/02/08. 10.1016/j.cell.2011.01.019 . 21295704

[7] Clapier CR, Cairns BR. The biology of chromatin remodeling complexes. Annu Rev Biochem. 2009;78:273–304. Epub 2009/04/10. 10.1146/annurev.biochem.77.062706.153223 . 19355820

[8] Gaillard H, Fitzgerald DJ, Smith CL, Peterson CL, Richmond TJ, Thoma F. Chromatin remodeling activities act on UV-damaged nucleosomes and modulate DNA damage accessibility to photolyase. J Biol Chem. 2003;278(20):17655–63. Epub 2003/03/15. 10.1074/jbc.M300770200 . 12637512

[9] Downs JA, Lowndes NF, Jackson SP. A role for Saccharomyces cerevisiae histone H2A in DNA repair. Nature. 2000;408(6815):1001–4. Epub 2001/01/05. 10.1038/35050000 . 11140636

[10] Chai B, Huang J, Cairns BR, Laurent BC. Distinct roles for the RSC and Swi/Snf ATP-dependent chromatin remodelers in DNA double-strand break repair. Genes Dev. 2005;19(14):1656–61. Epub 2005/07/19. 10.1101/gad.1273105 . 16024655 PMC1176001

[11] Gao F, Zhou BJ, Li GY, Jia PS, Li H, Zhao YL, et al A glutamic acid-rich protein identified in Verticillium dahliae from an insertional mutagenesis affects microsclerotial formation and pathogenicity. PLoS One. 2010;5(12):e15319 Epub 2010/12/15. 10.1371/journal.pone.0015319 . 21151869 PMC2998422

[12] Klosterman SJ, Atallah ZK, Vallad GE, Subbarao KV. Diversity, Pathogenicity, and Management of Verticillium Species. Annual Review of Phytopathology. 2009;47(1):39–62. 10.1146/annurev-phyto-080508-081748 19385730

[13] Wang S, Xing H, Hua C, Guo HS, Zhang J. An Improved Single-Step Cloning Strategy Simplifies the Agrobacterium tumefaciens-Mediated Transformation (ATMT)-Based Gene-Disruption Method for Verticillium dahliae. Phytopathology. 2016;106(6):645–52. Epub 2016/01/19. 10.1094/PHYTO-10-15-0280-R . 26780432

[14] Zhao Y-L, Zhou T-T, Guo H-S. Hyphopodium-Specific VdNoxB/VdPls1-Dependent ROS-Ca2+ Signaling Is Required for Plant Infection by Verticillium dahliae. PLOS Pathogens. 2016;12(7):e1005793 10.1371/journal.ppat.1005793 27463643 PMC4962994

[15] Zhou T-T, Zhao Y-L, Guo H-S. Secretory proteins are delivered to the septin-organized penetration interface during root infection by Verticillium dahliae. PLOS Pathogens. 2017;13(3):e1006275 10.1371/journal.ppat.1006275 28282450 PMC5362242

[16] Hemetsberger C, Herrberger C, Zechmann B, Hillmer M, Doehlemann G. The Ustilago maydis effector Pep1 suppresses plant immunity by inhibition of host peroxidase activity. PLoS Pathog. 2012;8(5):e1002684 Epub 2012/05/17. 10.1371/journal.ppat.1002684 . 22589719 PMC3349748

[17] O’Brien JA, Daudi A, Butt VS, Bolwell GP. Reactive oxygen species and their role in plant defence and cell wall metabolism. Planta. 2012;236(3):765–79. Epub 2012/07/07. 10.1007/s00425-012-1696-9 . 22767200

[18] Park CH, Chen S, Shirsekar G, Zhou B, Khang CH, Songkumarn P, et al The Magnaporthe oryzae effector AvrPiz-t targets the RING E3 ubiquitin ligase APIP6 to suppress pathogen-associated molecular pattern-triggered immunity in rice. Plant Cell. 2012;24(11):4748–62. Epub 2012/12/04. 10.1105/tpc.112.105429 . 23204406 PMC3531864

[19] He M, Xu Y, Chen J, Luo Y, Lv Y, Su J, et al MoSnt2-dependent deacetylation of histone H3 mediates MoTor-dependent autophagy and plant infection by the rice blast fungus Magnaporthe oryzae. Autophagy. 2018;14(9):1543–61. Epub 2018/06/23. 10.1080/15548627.2018.1458171 . 29929416 PMC6135590

[20] Li YB, Han LB, Wang HY, Zhang J, Sun ST, Feng DQ, et al The Thioredoxin GbNRX1 Plays a Crucial Role in Homeostasis of Apoplastic Reactive Oxygen Species in Response to Verticillium dahliae Infection in Cotton. Plant Physiol. 2016;170(4):2392–406. Epub 2016/02/13. 10.1104/pp.15.01930 . 26869704 PMC4825149

[21] Lundin C, North M, Erixon K, Walters K, Jenssen D, Goldman AS, et al Methyl methanesulfonate (MMS) produces heat-labile DNA damage but no detectable in vivo DNA double-strand breaks. Nucleic Acids Res. 2005;33(12):3799–811. Epub 2005/07/13. 10.1093/nar/gki681 . 16009812 PMC1174933

[22] Czaja W, Mao P, Smerdon MJ. Chromatin remodelling complex RSC promotes base excision repair in chromatin of Saccharomyces cerevisiae. DNA Repair (Amst). 2014;16:35–43. Epub 2014/03/29. 10.1016/j.dnarep.2014.01.002 . 24674626 PMC4026264

[23] Gelbart ME, Bachman N, Delrow J, Boeke JD, Tsukiyama T. Genome-wide identification of Isw2 chromatin-remodeling targets by localization of a catalytically inactive mutant. Genes Dev. 2005;19(8):942–54. Epub 2005/04/19. 10.1101/gad.1298905 . 15833917 PMC1080133

[24] Tackett AJ, Dilworth DJ, Davey MJ, O’Donnell M, Aitchison JD, Rout MP, et al Proteomic and genomic characterization of chromatin complexes at a boundary. J Cell Biol. 2005;169(1):35–47. Epub 2005/04/13. 10.1083/jcb.200502104 . 15824130 PMC2171912

[25] Hota SK, Bhardwaj SK, Deindl S, Lin YC, Zhuang X, Bartholomew B. Nucleosome mobilization by ISW2 requires the concerted action of the ATPase and SLIDE domains. Nat Struct Mol Biol. 2013;20(2):222–9. Epub 2013/01/22. 10.1038/nsmb.2486 . 23334290 PMC3565048

[26] Nakanishi S, Prasad R, Wilson SH, Smerdon M. Different structural states in oligonucleosomes are required for early versus late steps of base excision repair. Nucleic Acids Res. 2007;35(13):4313–21. Epub 2007/06/20. 10.1093/nar/gkm436 . 17576692 PMC1934998

[27] Whitehouse I, Rando OJ, Delrow J, Tsukiyama T. Chromatin remodelling at promoters suppresses antisense transcription. Nature. 2007;450(7172):1031–5. Epub 2007/12/14. 10.1038/nature06391 . 18075583

[28] Iida T, Araki H. Noncompetitive counteractions of DNA polymerase epsilon and ISW2/yCHRAC for epigenetic inheritance of telomere position effect in Saccharomyces cerevisiae. Mol Cell Biol. 2004;24(1):217–27. Epub 2003/12/16. 10.1128/MCB.24.1.217-227.2004 . 14673157 PMC303358

[29] Lan L, Ui A, Nakajima S, Hatakeyama K, Hoshi M, Watanabe R, et al The ACF1 complex is required for DNA double-strand break repair in human cells. Mol Cell. 2010;40(6):976–87. Epub 2010/12/22. 10.1016/j.molcel.2010.12.003 . 21172662

[30] Mathew V, Pauleau AL, Steffen N, Bergner A, Becker PB, Erhardt S. The histone-fold protein CHRAC14 influences chromatin composition in response to DNA damage. Cell Rep. 2014;7(2):321–30. Epub 2014/04/08. 10.1016/j.celrep.2014.03.008 . 24703848

[31] Dang W, Kagalwala MN, Bartholomew B. The Dpb4 subunit of ISW2 is anchored to extranucleosomal DNA. J Biol Chem. 2007;282(27):19418–25. Epub 2007/05/11. 10.1074/jbc.M700640200 . 17491017

[32] Parlanti E, Locatelli G, Maga G, Dogliotti E. Human base excision repair complex is physically associated to DNA replication and cell cycle regulatory proteins. Nucleic Acids Res. 2007;35(5):1569–77. Epub 2007/02/10. 10.1093/nar/gkl1159 . 17289756 PMC1865045

[33] Spiga MG, D’Urso G. Identification and cloning of two putative subunits of DNA polymerase epsilon in fission yeast. Nucleic Acids Res. 2004;32(16):4945–53. Epub 2004/09/25. 10.1093/nar/gkh811 . 15388803 PMC519108

[34] Ohya T, Maki S, Kawasaki Y, Sugino A. Structure and function of the fourth subunit (Dpb4p) of DNA polymerase epsilon in Saccharomyces cerevisiae. Nucleic Acids Res. 2000;28(20):3846–52. Epub 2000/10/12. 10.1093/nar/28.20.3846 . 11024162 PMC110797

[35] Zhou BJ, Jia PS, Gao F, Guo HS. Molecular characterization and functional analysis of a necrosis- and ethylene-inducing, protein-encoding gene family from Verticillium dahliae. Mol Plant Microbe Interact. 2012;25(7):964–75. Epub 2012/03/15. 10.1094/MPMI-12-11-0319 . 22414440

[36] Zhao P, Zhao YL, Jin Y, Zhang T, Guo HS. Colonization process of Arabidopsis thaliana roots by a green fluorescent protein-tagged isolate of Verticillium dahliae. Protein Cell. 2014;5(2):94–8. Epub 2014/02/01. 10.1007/s13238-013-0009-9 . 24481631 PMC3956967

[37] Zhang T, Zhao YL, Zhao JH, Wang S, Jin Y, Chen ZQ, et al Cotton plants export microRNAs to inhibit virulence gene expression in a fungal pathogen. Nat Plants. 2016;2(10):16153 Epub 2016/09/27. 10.1038/nplants.2016.153 . 27668926

[38] Jin Y, Zhao J-H, Zhao P, Zhang T, Wang S, Guo H-S. A fungal milRNA mediates epigenetic repression of a virulence gene in Verticillium dahliae. Philosophical Transactions of the Royal Society B: Biological Sciences. 2019;374(1767):20180309 10.1098/rstb.2018.0309 30967013 PMC6367151

[39] Bespalov VA, Conconi A, Zhang X, Fahy D, Smerdon MJ. Improved method for measuring the ensemble average of strand breaks in genomic DNA. Environmental and Molecular Mutagenesis. 2001;38(2–3):166–74. 10.1002/em.1068 11746751

[40] L M S. Pyrogallol autoxidation In: Greenwald RA, editor. Handbook for oxygen radical research. Boca Raton: CRC Press; 1985 p. 243–7.

