# Supplementary figures and images for: Verticillium dahliae chromatin remodeling facilitates the DNA damage repair in response to plant ROS stress
Source: PLoS Pathog. 2020 Apr 16;16(4):e1008481. doi: 10.1371/journal.ppat.1008481 (PMC7188298; doi:10.1371/journal.ppat.1008481)

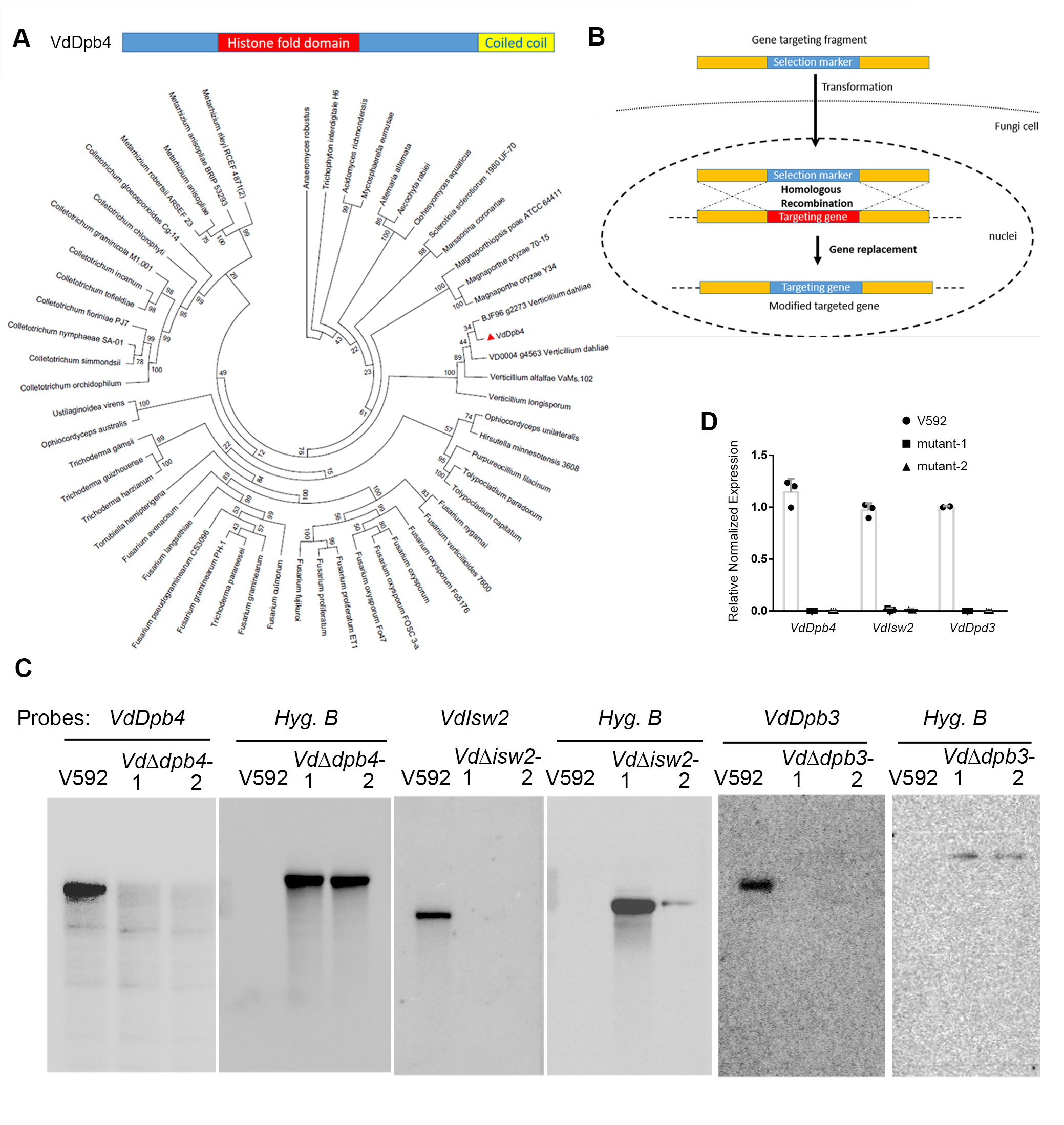

Supplement: S1 Fig — A. Schematic representation of the VdDpb4 protein comparison of the histone-fold motif and coiled-coil domain. Phylogenic analysis of the VdDbp4 homologs with other Dpb4 proteins available from a limited number of fungal species as indicated. VdDbp4 is labeled. The analysis was performed using the neighbor-joining method phylogeny test with the bootstrap method (No. of bootstrap replications = 1000). B. Schematic representation of the homologous recombination event involved in the targeted replacement of fungal gene. C. Southern blot analysis of targeted gene deletion mutants. Hind III digested genomic DNA from V592 wild type strain and two putative of VdΔdpb4, VdΔisw2, and VdΔdpb3 transformants were blotted with the probe indicated on the top of gels. D. Reverse transcription qPCR (RT-qPCR) analysis of the expression of VdDpb4, VdIsw2 and VdDpb3 in knockout mutants with primers listed in S1 Table. (TIF) [file ppat.1008481.s001.tif]

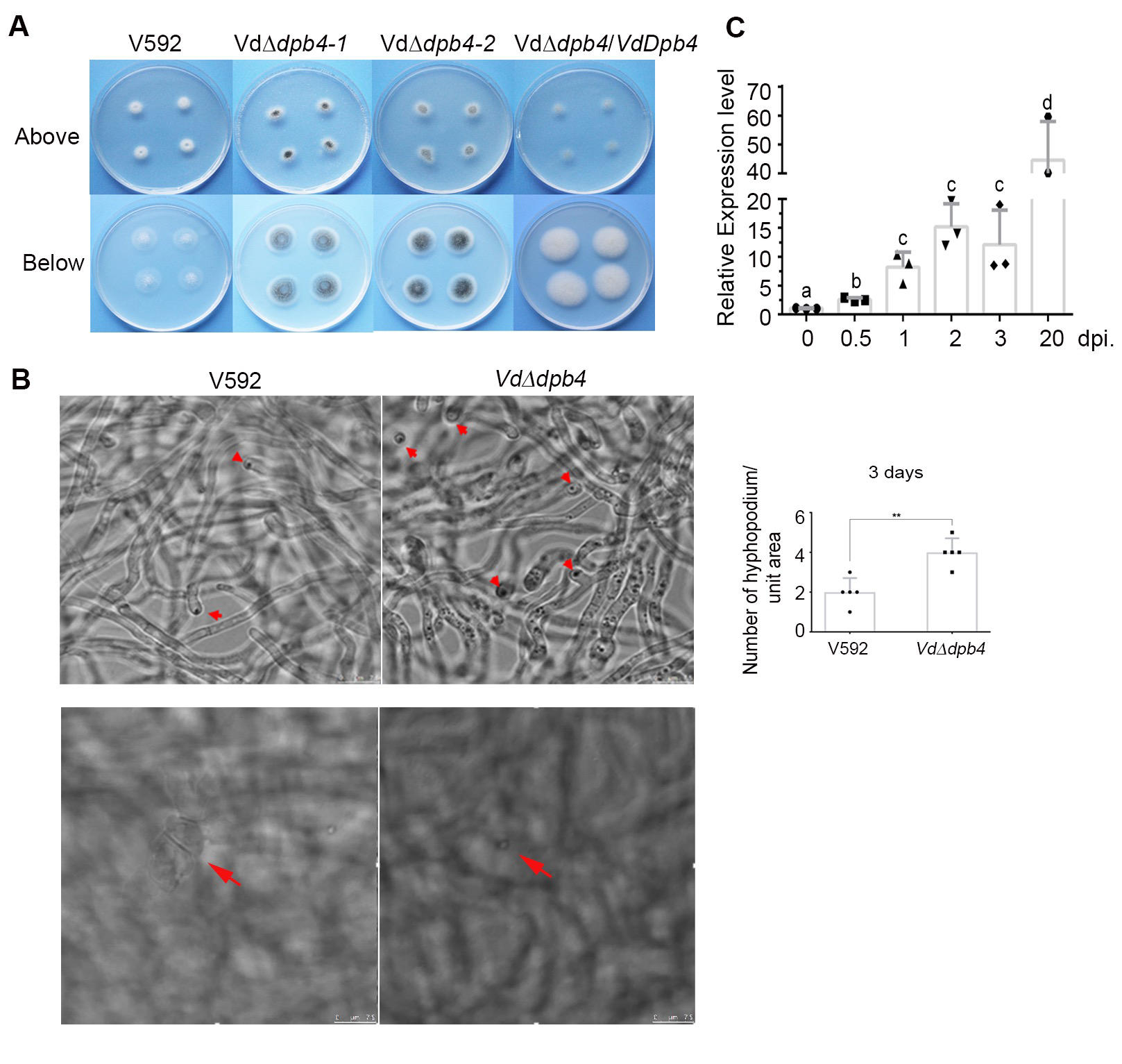

Supplement: S2 Fig — A. Penetration assay with a cellophane membrane. Colonies of V592, the VdΔdpb4 mutant strains, and the VdΔdpb4/VdDpb4 complementation strains grown on MM medium overlaid with a cellophane layer (above) and removal of the cellophane membrane (below). Photographs in the first row were taken at 3 dpi. The second row shows growth of V592, the VdΔdpb4 mutant strains, and the VdΔdpb4/VdDpb4 complementation strains on MM medium after penetration of the cellophane membrane. B. Statistical analysis of the hyphopodia on the cellophane membrane at 3 dpi. Differentiation of hyphopodia (swollen hyphae) in V592 and VdΔdpb4 is indicated by arrows. More than three areas were counted by random selection, and the average number of hyphopodia was calculated. Error bars represent standard deviations. Hyphopodium could penetrate the cellophane membrane and grow under the membrane as showed and indicated with arrows. Asterisks indicate significant differences (P < 0.05, t-test, mean ± SD). C. VdDpb4 expression was rapidly induced at early time points during cotton infection as detected by RT-qPCR). Different letters indicate significant differences of gene expression at P< 0.05, mean ± SD, one-way analysis of variance (ANOVA) followed by Tukey’s multiple comparisons test. (TIF) [file ppat.1008481.s002.tif]

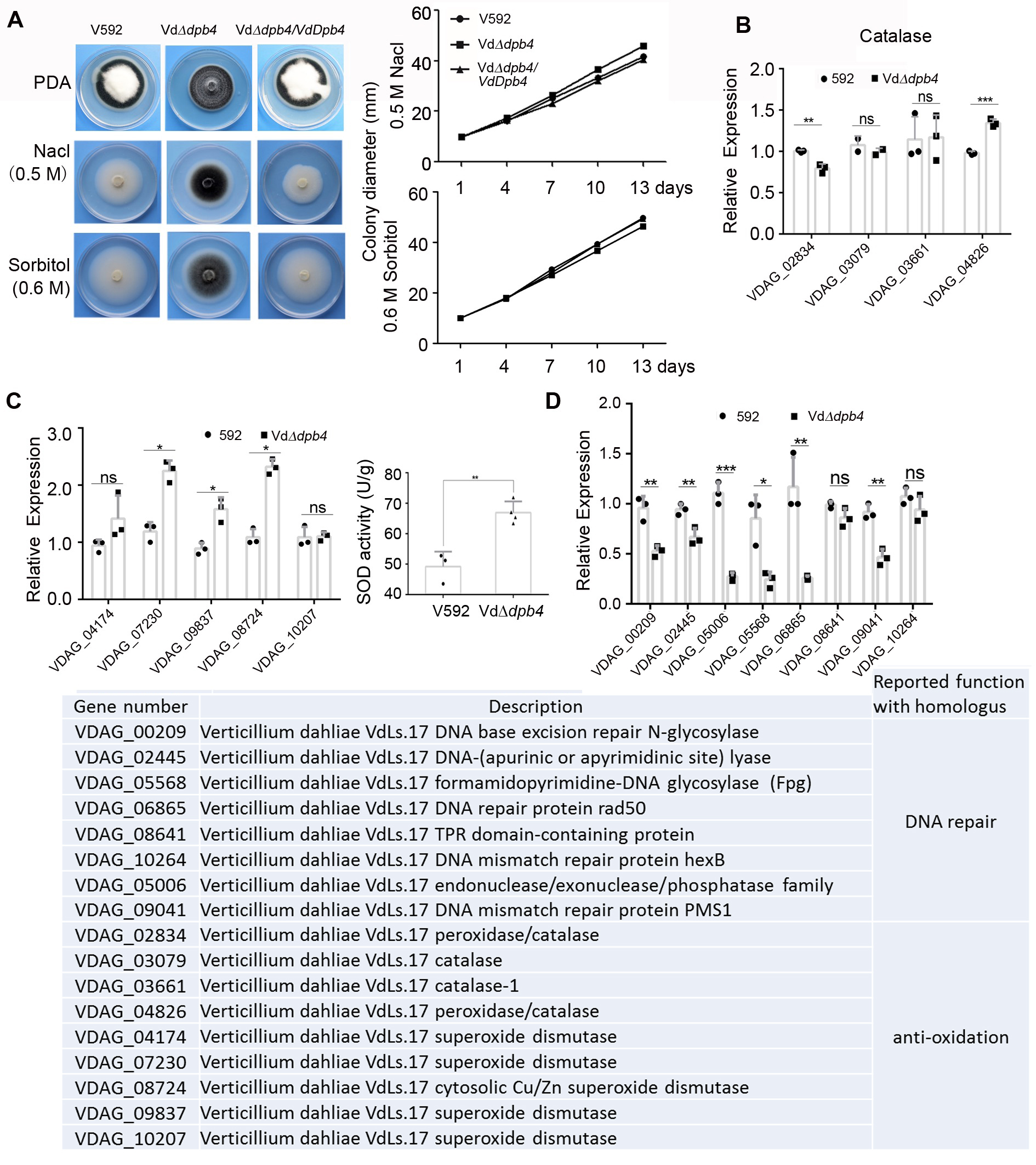

Supplement: S3 Fig — A. Mycelial growth on PDA agar medium with NaCl and sorbitol and quantification of colony diameter. B. RT-qPCR analysis of the expression level of the catalase-encoding genes in the V592 and VdΔdpb4 mutant strains. Error bars represent standard deviations. C. RT-qPCR analysis of the expression level of the SOD-encoding genes in the V592 and VdΔdpb4 mutant strains. Asterisks indicate significant differences (P<0.05; t-test, mean ± SD). D. RT-qPCR analysis of the expression level of the genes involved in DNA damage repair. Asterisks indicate significant differences (P<0.05; t-test, mean ± SD). (for B-D, the name description and function of the genes analyzed were listed below). (TIF) [file ppat.1008481.s003.tif]

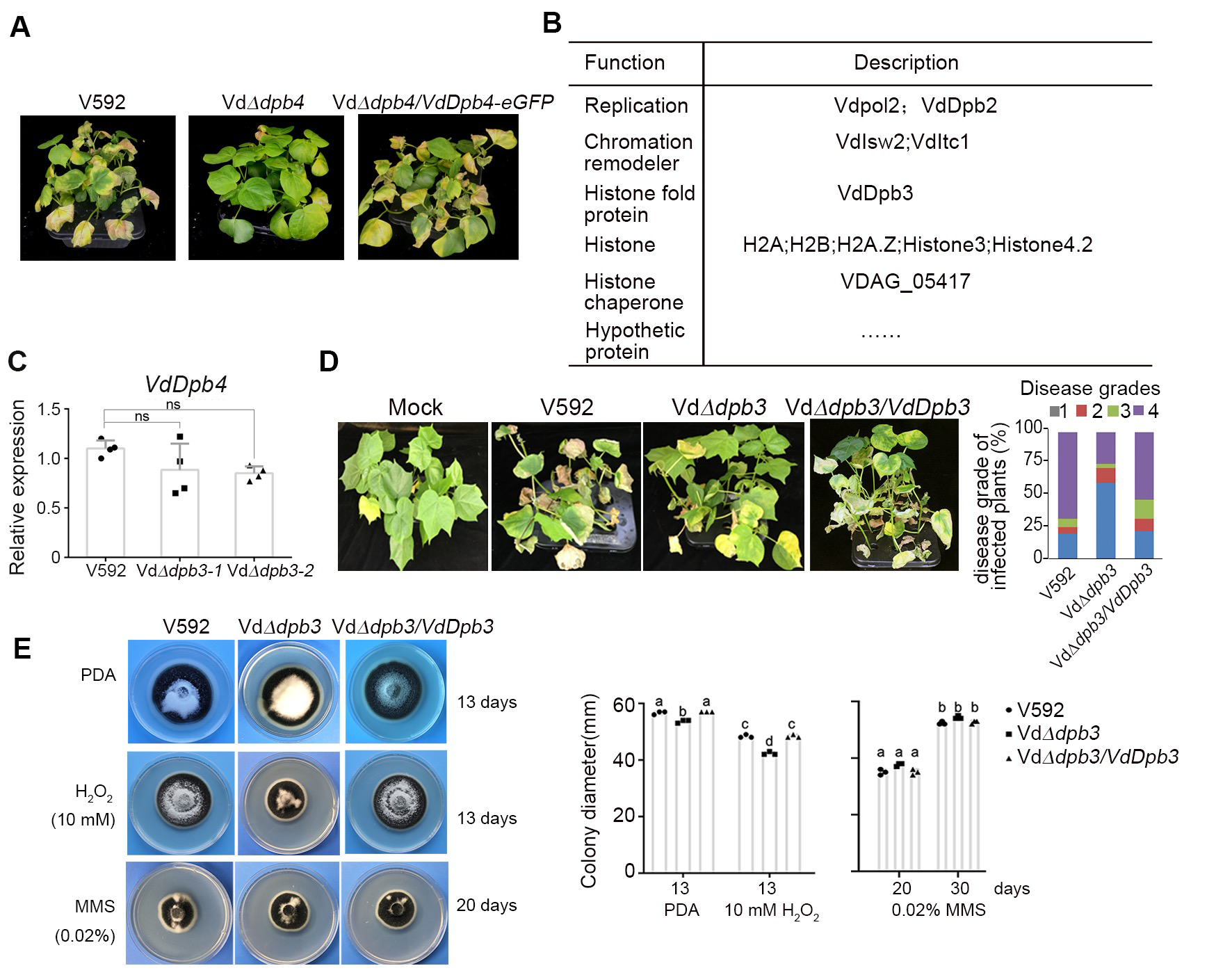

Supplement: S4 Fig — A. VdDpb4-eGFP expression in VdΔdpb4 mutant restored virulence of the mutant in cotton plants. B. The proteins identified by mass spectrometry analysis of purified VdDpb4 were grouped on the basis of their functions. Full list of proteins identified is shown in S2 Table. C. Expression of VdDpb4 in V592 and VdΔdpb3 mutants. (ns: no significant difference, t-test, mean ± SD). D. Disease symptoms of cotton plants infected with V592 or VdΔdpb3 at 30dpi. Disease grades on cotton leaves were classified into four levels dependent on the ratio of (wilted and dropped off leaves / total leaves) during fungal invasion: grade 1 = 0–25%; grade 2 = 26–50%; grade 3 = 51–75%; and grade 4 = 76–100%. E. Quantification of colony diameter cultured on PDA media with H2O2 and MMS. Different letters indicate significant differences of fungal growth at P< 0.05, mean ± SD, one-way analysis of variance (ANOVA) followed by Tukey’s multiple comparisons test). (TIF) [file ppat.1008481.s004.tif]

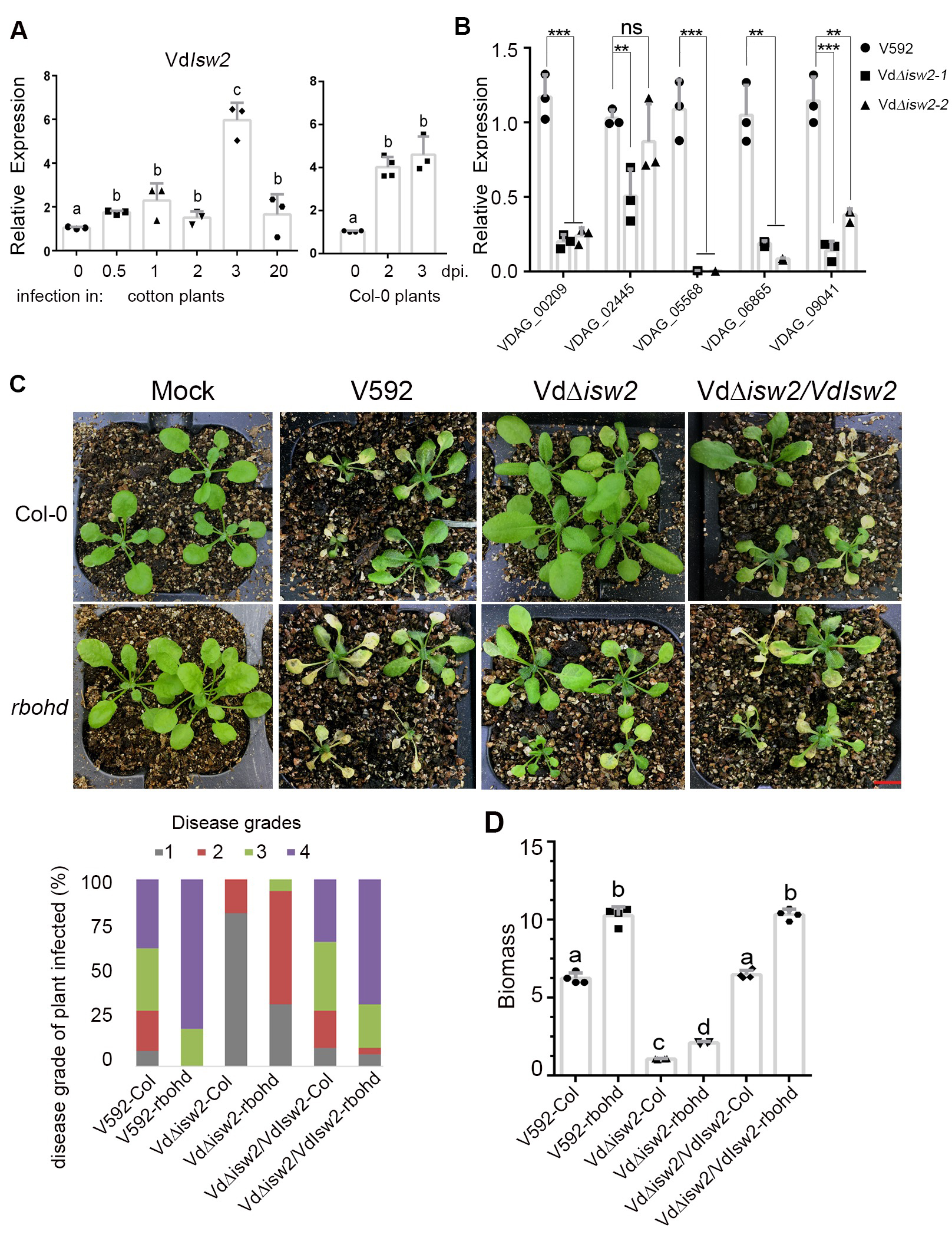

Supplement: S5 Fig — A. VdIsw2 expression was induced at early time points during cotton and Arabidopsis plant infection as detected by quantitative RT-PCR (RT-qPCR). Different letters indicate significant differences of gene expression at P< 0.05, mean ± SD, one-way analysis of variance (ANOVA) followed by Tukey’s multiple comparisons test). B. RT-qPCR analysis of the expression level of genes involved in DNA damage repair (gene names were shown in S3 Fig). Asterisks indicate significant differences, ns: no significant difference, (P<0.05; t-test, mean ± SD). C. VdIsw2 is essential for resistance to RBOHD-mediated defense. Disease symptoms of wild-type (Col-0) and rbohd mutant Arabidopsis plants infected with V592, mutant or complementation strains at 20 dpi. The disease grades were evaluated with three replicates of 48 plants for each inoculum. D. Reduced fungal biomass in VdΔisw2-infected Arabidopsis plants compared with V592-infected ones at 2-week postinoculation. The values were quantitative real time (qPCR) of fungal tubulin DNA relative to Arabidopsis At4g33380 DNA. Statistical analysis was described as in A. (TIF) [file ppat.1008481.s005.tif]

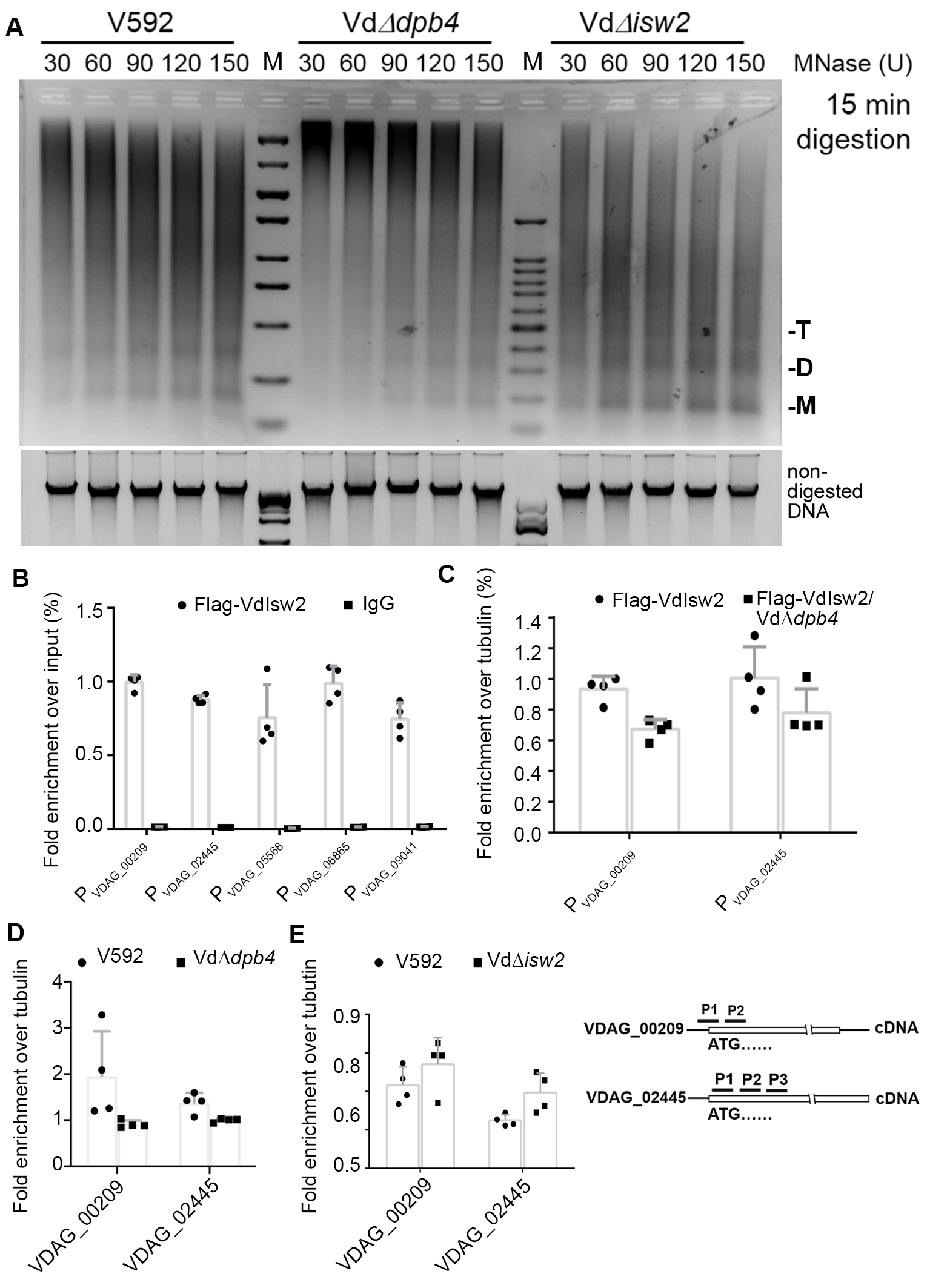

Supplement: S6 Fig — A. MNase digestion patterns in the wild-type V592, VdΔdpb4 and VdΔisw2 mutant cells synchronized at the G2/M phase of the cell cycle. The gel shows an experiment with 15 min MNase digestion. M: DNA size standard, T: trinucleosome, D: dinucleosome, M: mononucleosome. B. ChIP-qPCR analysis showing that VdIsw2 could bind to the gene promoter region involved in DNA damage repair. ChIP assays were conducted in cells expressing Flag-VdIsw2 using an anti-Flag antibody. C. ChIP-qPCR analysis showing that VdIsw2 binding to the gene promoter region was reduced in the Flag-VdIsw2/VdΔdpb4 mutant strain compared with the wild-type Flag-VdIsw2 strain. ChIP assays were conducted in cells expressing Flag-VdIsw2 using an anti-Flag antibody. Tubulin was used as a control. D. ChIP-qPCR analysis showing that the recruitment of RNA polymerase II was reduced in the VdΔdpb4 mutant strain compared with the wild-type V592 strain. ChIP assays were conducted with an anti-Pol II antibody. Tubulin was used as a control. E. ChIP-qPCR analysis showing little difference in the recruitment of pol II to the tested DNA repair genes in the VdΔisw2 mutant strain compared with the wild-type V592 strain. Tubulin was used as a control. (For B-E, gene name and description were in S3 Fig. For D-E, a scheme at right shows where the oligonucleotides used in each gene. (TIF) [file ppat.1008481.s006.tif]
