# Supplementary material for: Verticillium dahliae chromatin remodeling facilitates the DNA damage repair in response to plant ROS stress
Source: PLoS Pathog. 2020 Apr 16;16(4):e1008481. doi: 10.1371/journal.ppat.1008481 (PMC7188298; doi:10.1371/journal.ppat.1008481)
Supplement: S1 Table — (PDF) [file ppat.1008481.s007.pdf]

Primer list in this study

Primer name	Sequence(5'-3')	function
VdELF-s	CCATTGATATCGCACTGTGG	QPCR-reference
VdELF-a	TGGAGATACCAGCCTCGAAC	
β-tublin-s	AgCTCACCCAgCAgATgTTC	
β-tublin-a	TCgACCTCCTTCATggCAAC	
18srRNA-s	CCGCCGGTCCATCAGTCTCTCTGTTTATA	
18srRNA-a	CGCCTGCGGGACTCCGATGCGAGCTGTAAC	
0537up-up-s	CAATGCACGTTCCAGGGCGACC	Knockout
0537up-s	GGGTTTAAUGTTTACCAATCTTGCTTCCCGC	
0537up-a	GGACTTAAUAAGCGGTGGGACTGAGAGAG	
0537dn-s	ACTGCTGGCCGGATCCATGACGGGCTAGTGGCCAGTT	
0537dn-a	TAGAACTAGTGGATCCCTGACAAGACAGCCCCACGACT	
0537dn-dn-a	CTTCCCTTCCTTCTGCTCTGCC	
1523up-up-s	CCGTCAGTGATGTCTCGC	
1523up-s	GGGTTTAAUACAGGTCTGGAACGCCCGAAGG	
1523up-a	GGACTTAAUATTCGCTTTACGCGACTGCGAC	
1523dn-s	GGCATTAAUCAAGCCTGGATCCGAGAAGTTC	
1523dn-a	GGTCTTAAUTGCTCTCATAGTCTGTCCTGCC	
1523dn-dn-a	GAAAACCTTGGCGTATTGCTTG	
1523(215K-R)mutation-s	gccgatgaaatgggtcttggcCGAacattacagacgatatccttcctc	Isw2 mutantion
1523(215K-R)mutation-a	gaggaaggatatcgtctgtaatgtTCGgccaagacccatttcatcggc	
NEO2TEF-8781-VN-S	ACCGTCAAACGGATCCATGCCGCCCAAAAAGCCCTCACC	BIFC
NEO2TEF-8781-VN-A	CGCCGGCGCCGGATCCATCACTGTCATTGCCATCCAGC	
NEO2TEF-VN-8781-S	CGCCGGCGCCGAATTCATGCCGCCCAAAAAGCCCTCACC	
NEO2TEF-VN-8781-A	AACGTTAAGTGAATTCTTAATCACTGTCATTGCCATCCAGC	
HPTOlicP-0537-VC-S	TCACATCACAGGATCCATGCCCTACAACACCACAGCCA	
HPTOlicP-0537-VC-A	CGCCGGCGCCGGATCCTGTCGACGTCGCCATCGTG	
NEO2TEF-1523-VN-S	ACCGTCAAACGGATCCATGCCTCCCTCAAGACCACGC	
NEO2TEF-1523-VN-A	CGCCGGCGCCGGATCCCTTCTTTTTCCCCTTGGCTTTGGA	
HPTOlicP-3720-VC-S	TCACATCACAGGATCCATGGTTCTTTTCAAGCGAAAGCC	
HPTOlicP-3720-VC-A	CGCCGGCGCCGGATCCCTTCTTCCGCCTCGTCTTGGGAG	
NEO2TEF-VN-3720-S	CGCCGGCGCCGAATTCATGGTTCTTTTCAAGCGAAAGCC	
NEO2TEF-VN-3720-A	AACGTTAAGTGAATTCCTTCTTCCGCCTCGTCTTGGGAG	
NEO2TEF-VN-1523-S	CGCCGGCGCCGAATTCATGCCTCCCTCAAGACCACGC	
NEO2TEF-VN-1523-A	AACGTTAAGTGAATTCTTACTTCTTTTTCCCCTTGGCTTTGGA	
HPTOlicP-1523-VC-S	TCACATCACAGGATCCATGCCTCCCTCAAGACCACGC	
HPTOlicP-1523-VC-A	CGCCGGCGCCGGATCCCTTCTTTTTCCCCTTGGCTTTGGA	
VC-5878-S	CGCCGGCGCCGAATTCGCAGAGGAACCGTACGCGCC	
VC-5878-A	CGCGGCCGCTGAATTCTCACGTGGCTGCCTTCTTCT	
BIFC-VC-1639-s	TCACATCACAGGATCCATGTATCACAAAAAACCACAGCG	
BIFC-VC-1639-A	CGCGGCCGCTGAATTCTCACGACGACCGCTTCAGGTCGAC	
BIFC-VC-8774-s	TCACATCACAGGATCCATGAACTCCCTACCTGACGGTGA	
BIFC-VC-8774-s	CGCGGCCGCTGAATTCTCAATTAATAAGCCCCTCCATCCCCC	
CHIP0209-1S	CCGTCAGACTGGGAAGAGATG	
CHIP0209-1A	ACCGTGTCTTTTGTCTGGCT	
CHIP0209-2S	AAAGTCAGTCTCTGGCCCCT	
CHIP0209-2A	TCGTGGATCAATCCGAGGGT	
CHIP0209-3S	CTCTGCCCCATCTGCCTTAC	
CHIP0209-3A	TGAACCTGGGGCTGTTTAGC	
CHIP0209-4S	TGAAAGAAGACGACGTGCCC	

CHIP0209-4A	TCATGTATCGCTTCCCGCTT	
CHIP2445-1S	ACGGTGAGTCGTGTCTGAAC	PolII chip
CHIP2445-1A	TCAGCCAACCTCGAATGTCC	
CHIP2445-2S	TCGAGGTTGGCTGACACATC	
CHIP2445-2A	CAGCCGGGAATGAGAACCAT	
CHIP2445-3S	ATGCAACGTGCTGTCCCATA	
CHIP2445-3A	GGGACTGTACGTGCCAATGA	
CHIP2445-4S	CCTGCATTCGTGCTCATTGG	
CHIP2445-4A	GGATCGGCTGACGTTCAGAT	
CHIP2445-5S	GTGCCAGAGAGAAAGACGCT	
CHIP2445-5A	GTTGGGGAATGCAGGGGTAA	
QPCR4174Q1-S	CGCATCCTGACGACCTATC	
QPCR4174Q1-A	CTCCCACGTGTTGAGGCACA	
QPCR4174Q2-S	GCCCACAACACGCACTTCT	
QPCR4174Q2-A	GGAGGCCTTGACGAGCCAGA	
QPCR7230Q1-S	CCATTGTCACCACGAAGGA	
QPCR7230Q1-A	CCAGTTGATGACATTCCAGA	
QPCR8724Q1-S	ACCACATCCACGACTCTCCG	SOD
QPCR8724Q1-A	GATGAGAGACGAGTAAGGG	
QPCR8724Q2-S	GTCCTTCACTTCAGCAGCAA	
QPCR8724Q2-A	AGCAGTAGGCACGGGAACGA	
QPCR9837Q1-S	TCGTCAAGCAGGACGTCAGC	
QPCR9837Q1-A	CCAACAGTCCATCGAGCACA	
QPCR10207Q1-S	CAAGGACAAGACAAGTGGC	
QPCR10207Q1-A	TCTCAAAGCGGCTGGCAACG	
QPCR-05568-S	CATGAAACCAGAGGAAGCAG	
QPCR-05568-A	AATGTCGTCCCCGGGACAGT	
QPCR-01784-S	CCTGATCCTCTTCTGCCTCC	
QPCR-01784-A	TCGTCCACACTCTTTTCCAT	
QPCR-05006-S	CACCCTTATTCCCCTTCCAC	
QPCR-05006-A	TACCGACAACGACGTCCTTG	
QPCR-00209-S	ATCCTCATCTCCCTGATGAT	
QPCR-00209-A	TTGTTGTTGTGGAAACCCAC	
QPCR-06865-S	GACGATCTCCAATCCTGC	
QPCR-06865-A	TAGTCCCATTTCCCCACT	
QPCR-6065-S	CGACGATGGTTGGGAACA	
QPCR-6065-A	TCGAAAGGGACTCGACACG	
QPCR-09396-S	AGCCACTGCATGCTCTAC	
QPCR-09396-A	TCGCCAACTGACAAACTC	
QPCR-9966-S	GATCCACACCGCCATGTT	
QPCR-9966-A	TCCGTCGATTTCACCCTC	DNA repair pathway relative gene
QPCR-9866-S	CAAGACACTCGAAGGGGT	
QPCR-9866-A	TCTGGGTTGAGAAACAGC	
QPCR-9041-S	TATGCTCCCCCGTCTAAAA	
QPCR-9041-A	GGATCATGACCAGCGCTAT	
QPCR-8623-S	CTGGTCGAGGTCGTATGAT	
QPCR-8623-A	ACGGCGTGTTGTGTAAAGT	
QPCR-10264-S	TCACACGTTTGTCGGAATTG	
QPCR-10264-A	GACTGAAGCGTATGGAGCC	
QPCR-8641-S	CTGAAGAGTTTGGTGGGGT	
QPCR-8641-A	TGATGGTTGGACGGTGACA	
QPCR-00787-S	AGCATCCTGCTACCCTTCC	

QPCR-00787-A	CAGCTCTCCGTCCACAAAC	
QPCR-2445-S	ACAAGACCAGCCAATAACG	
QPCR-2445-A	GTGTCCCCCAAAGTACCAT	
QPCR-03983-S	CCGATTTTGCTTGCTGTCC	
QPCR-03983-A	TTGTATCACGCCGCTTCCT	
QPCR-1523-1S	TGATGACCGCATGGAGGTTGAC	infection
QPCR-1523-1A	CGGAGAGTGTCTTCTTCCTTTG	
QPCR-1523-2S	GTTTGAGGACGTTCAGCCAACC	
QPCR-1523-2A	CGAGCGCTTTGTTCTTGACTCC	
QPCR-8781-2S	CTCGCCACCCACGCAAACGAAC	
QPCR-8781-2A	TCTGGGCGTATTTCGCGAATTC	
QPCR00209PQ1-S	GCGTCATCAAAGGCGAGTC	Flag-Isw2 chip
QPCR00209PQ1-A	AGATGAGGATCTGGAAGCG	
QPCR2445PQ1-S	CCAAAGAGCGACAATCCAT	
QPCR2445PQ1-A	TGTTCTTGGCGTTACGGACC	
QPCR-02834-S	GGAGACCGTCGCTCTTATT	catalase
QPCR-02834-A	GACCCTTGCCAGACTTGTA	
QPCR-3079-S	GCAACCCGACCAACTTTCT	
QPCR-3079-A	CCCTCCTTGTTCACCCACT	
QPCR-3661-S	CCTACACCCTCATCAACGA	
QPCR-3661-A	GCAGCACCTGAATACCAAA	
QPCR-4826-S	TGACATCCGCACCAACTGG	
QPCR-4826-A	CGTGAGAGCCGAAAACGAG	
5792QPCR1-A	CCTCTGCTGTCTGTGGATT	
6575QPCR1-S	GGCATCCACAACAATAACC	
6575QPCR1-A	TCTCCGACCTCTCACGAAC	
9115QPCR1-S	CGAGTGCACCAACCCTATC	
9115QPCR1-A	TCCGTCCTGAACTTGACAG	
5878nativeP-NEO2-F	TGGCCTGTTGCTAACTAGTCGAAGACAGGACACGAGAGGCA	CoIP and complementation
5878-EK-R	CGGCAGCTTCTGCGAATTCCGTGGCTGCCTTCTTCTTGCG	
VdDpb4-NP-F	GACGGCCAGTGCCAAGCTTCTCTGTAGCGAAGCACACTCGGGC	
VdDpb4-NP-R	CGGCAGCTTCTGCGAATTCCTGTCATTGCCATCCAGCGCCTCGTC	
VdDpb4-NP-R（stop codon）	CGGCAGCTTCTGCGAATTCTCACTGTCATTGCCATCCAGCGCCTCGTC	
VdDpb3-NP-F	GACGGCCAGTGCCAAGCTTTGCTACTTTACGCTCATCAA	
VdDpb3-NP-R	CGGCAGCTTCTGCGAATTCTCATGTCGACGTCGCCATCGTG	
FLAG-1523-S	CCCGGGGGAGAATTCATGCCTCCCTCAAGACCACGC	
FLAG-1523-A	CGCGGCCGCTGAATTCCTTCTTTTTCCCCTTGGCTTTG	
EK-8781CDS-S	AACCTCTAGAGGATCCATGCCGCCCAAAAAGCCCTCAC	constituted promoter
EK-8781CDS-A	CACCAGCACCGAATTCATCACTGTCATTGCCATCCAGC	
